# Transcriptome Analysis of a Rotenone Model of Parkinsonism Reveals Complex I-Tied and -Untied Toxicity Mechanisms Common to Neurodegenerative Diseases

**DOI:** 10.1371/journal.pone.0044700

**Published:** 2012-09-07

**Authors:** Yofre Cabeza-Arvelaiz, Robert H. Schiestl

**Affiliations:** Departments of Pathology and Environmental Health Sciences, David Geffen School of Medicine and School of Public Health, University of California Los Angeles, Los Angeles, California, United States of America; National Institute of Health, United States of America

## Abstract

The pesticide rotenone, a neurotoxin that inhibits the mitochondrial complex I, and destabilizes microtubules (MT) has been linked to Parkinson disease (PD) etiology and is often used to model this neurodegenerative disease (ND). Many of the mechanisms of action of rotenone are posited mechanisms of neurodegeneration; however, they are not fully understood. Therefore, the study of rotenone-affected functional pathways is pertinent to the understanding of NDs pathogenesis. This report describes the transcriptome analysis of a neuroblastoma (NB) cell line chronically exposed to marginally toxic and moderately toxic doses of rotenone. The results revealed a complex pleiotropic response to rotenone that impacts a variety of cellular events, including cell cycle, DNA damage response, proliferation, differentiation, senescence and cell death, which could lead to survival or neurodegeneration depending on the dose and time of exposure and cell phenotype. The response encompasses an array of physiological pathways, modulated by transcriptional and epigenetic regulatory networks, likely activated by homeostatic alterations. Pathways that incorporate the contribution of MT destabilization to rotenone toxicity are suggested to explain complex I-independent rotenone-induced alterations of metabolism and redox homeostasis. The postulated mechanisms involve the blockage of mitochondrial voltage-dependent anions channels (VDACs) by tubulin, which coupled with other rotenone-induced organelle dysfunctions may underlie many presumed neurodegeneration mechanisms associated with pathophysiological aspects of various NDs including PD, AD and their variant forms. Thus, further investigation of such pathways may help identify novel therapeutic paths for these NDs.

## Introduction

Gene-environment interactions have been implicated in the etiology of neurodegenerative diseases (NDs) [1]–[3]. Rotenone, a flavonoid often used as a pesticide, is a neurotoxin that induces neurodegeneration. Indeed, chronic treatment of animals and in vitro NDs models of rotenone replicate certain features of Parkinson disease (PD) and Alzheimer disease (AD) including motor deficits, α-synuclein (SNCA) upregulation and aggregation, tau (MAPT) and amyloid β peptides (Aβ) accumulation, and dopaminergic and cholinergic cell death [4]–[10]; and chronic exposure to rotenone has been positively linked with PD [3]. The mechanisms of action of rotenone, leading to neuronal cells death in vivo and in vitro, involve increased oxidative stress (OS) [5], [11]–[15]; which was thought to be solely the result of mitochondrial complex I inhibition by rotenone [5], [16]. However, recent studies compellingly show that rotenone effects can be mediated independently of complex I inhibition [17], [18]. This neurotoxin has been shown to affect a variety of processes that include, besides mitochondria function and microtubule (MT) stability, Ca2+ homeostasis, OS, DNA damage response (DDR), proteasome function, inflammatory response and apoptosis [5], [11]–[14], [17]–[24]. All such studies used directed approaches focusing on a few of the genes/proteins involved; transcriptome analysis is an alternative approach for the detection of key changes that might not be practical to attempt by single-gene approaches. This report describes the results from such an analysis on an in vitro rotenone neurodegeneration model of PD [11]; modified by not using pyruvate, a known protector against rotenone neurotoxicity [25], [26], during the chronic exposure of human neuroblastoma (NB) cells to marginally toxic and moderately toxic doses of rotenone [11], [12], [21], [22]. The data support a response to rotenone that includes established and novel mechanisms; such as the complex I inhibition-independent enhancement of OS and energy depletion, possibly through the destabilization of the MT system and blockage of voltage-dependent anions channels (VDACs), leading to cell-cycle disruptions, promotion of differentiation and neuroprotection, and the activation of apoptotic pathways.

## Results and Discussion

### Rotenone Toxicity and Effects on Proliferation are Dose and Time-dependent

**Figure 1:**
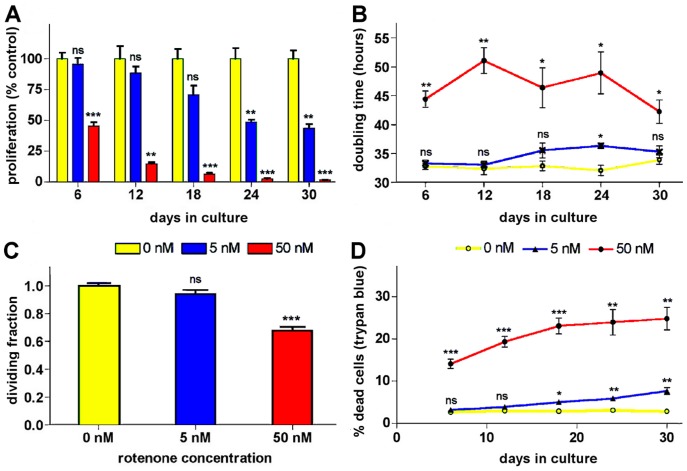
Proliferation and cytotoxicity analysis. Different proliferation rates of exponentially growing cell populations exposed to increasing amounts of rotenone over a 30-day period. Growth kinetic data (see **Fig. S3**) of rotenone-treated and vehicle-treated (control) cells was used to determine: (**A**) the cell proliferation rates, expressed as a percentage of the average growth rate of the treated cells relative to the control cells (assumed to grow at 100% rate), (**B**) the cell population doubling times, and (**C**) the fraction of dividing cells or mitotic fraction. (**D**) rotenone cytotoxicity was assayed, at each time point, by determining the percentage of dead cells under each treatment condition using the trypan blue exclusion method. Data are presented as mean ± S.E.M of the respective measured parameters at each time point (N = 3); except for the dividing fractions in C, which is expressed as the average of values from all time points (N = 5), * P<0.05, ** P<0.005 and *** P<0.005 compared with the respective vehicle-treated control.

Reported IC50 for rotenone ranges between 200 µM and 20 nM depending on the cell type [18], [27], [28] and primary neurons reported IC50 for rotenone is 20 nM [18]; the human NB SK-N-MC cells, with an IC50 of 20–30 nM [11], are as sensitive to rotenone as primary neurons. In this study we investigated the effects of rotenone doses, lower (5 nM) and higher (50 nM) than the IC50 in SK-N-MC, on gene expression during chronic short (1 week) and prolonged (4 weeks) exposures. However, prior to performing the transcriptome analysis studies the relative toxicity of such rotenone doses was ascertained by assaying their effects on SK-N-MC cells proliferation and death. The proliferation levels under each treatment, relative to that of untreated cells (assumed as 100%), shown in **Fig. 1A**, illustrate the time-dose-dependent cumulative effect of rotenone on cell growth; which becomes significant with the lower dose only after 3 weeks. Noteworthy, such an effect by the 5 nM dose seems to vanish when 5 mM pyruvate is used; as no effect on cell growth kinetics was seen with this dose [29]; even though, ∼5% apoptosis was detected at 4 weeks [11], [29]. The cell populations doubling times (PDT) shown in **Fig. 1B** also suggest that rotenone effects on proliferation fluctuate with time. As the SK-N-MC cell line is an intermediate type (I-type) of NB cells [30], with properties of both the neuron-like neuroblastic (N) type and the glial-cells-like, substrate adherent (S) type, that can transdifferentiate into both S- and N-type cells [30]–[34]; such fluctuations could be due to differential response to rotenone by the different cell types. The decreased PDTs after 4 weeks, particularly with the higher dose, may reflect adaptation or rotenone tolerance by transcriptional regulation as described below. The fraction of non-dividing cells, estimated as ∼6% and 32% for the 5 nM and 50 nM doses, respectively (**Fig. 1C**), was seemingly not significantly different from the control with the lower dose. Though, such value is close to the 5% apoptosis previously detected with the 5 nM dose and pyruvate supplementation [11], which suggests that apoptotic events independent of pyruvate depletion contribute to the effect of the lower dose on proliferation as seen **Fig. 1A**. However, this does not preclude contributions from alterations in glycolysis; as the PDT formula used here does not account for the fraction of non-dividing cells due to cell cycle arrest, differentiation, or cell death, and thus the calculated values are likely underestimated. A more accurate assessment would entail measuring the fractions of mitotic, differentiating and dying cells by various techniques [35], not feasible with prolonged exposure studies. Indeed, the measured rotenone cytotoxicity levels, (**Fig. 1D**), revealed a moderate toxicity (∼25% cell death) with the higher dose and a restrained (∼8% cell death) but significant toxicity with the lower dose. Though, such levels may also be underestimated due to caveats of the method used; such as the inability to score cells in late apoptosis that detach and burst in the medium. A death percentage closer to 50% with 50 nM rotenone, determined by daily scoring of apoptotic cells or decreased total protein for 5 days, has been reported [12]. Nonetheless, the results suggest that the 5 nM dose is marginally toxic while the 50 nM dose is moderately toxic.

### Rotenone-induced Gene Expression Changes are Dose and Time-dependent

Gene expression was monitored in the context of an in vitro PD model in SK-N-MC cells exposed for 1 and 4 weeks to the 5 nM or the 50 nM dose of rotenone. Quality control (QC) assessment of the data from the transcriptome analysis revealed the high quality of the data (see **Table S1**). As indicated by the number of present calls, at or above 59% in all replicates, with no significant difference (P<0.05) among them; and by the median signal intensity that was ∼3 times the background level and similar in all samples. A summary of the number of differentially-regulated genes (DRGs) in the most prominently impacted cellular processes is given in **Table 1**, and the complete list of DRGs is shown in **Table S2**. Gene symbols in this report are those used in the Gene database at the National Center for Biotechnology Information (http://www.ncbi.nlm.nih.gov/gene/).

**Table 1:** Enriched functional categories for rotenone differentially-regulated genes (DRGs).

Group	GO functional category	DRGs (at 1 week)		DRGs (at 4 weeks)	
#	(enriched GO terms)	5 nM^c^	50 nM^c^	5 nM^c^	50 nM^c^
		up/down	up/down	up/down	up/down
1	regulation of apoptosis/GO:0042981	9/1	9/5	**22/22**	**27/34**
1	− ve regulation of apoptosis/GO:0043066	*4/1*	5/3	**11/12**	**12/24**
1	+ ve regulation of apoptosis/GO:0043065	*5/0*	*4/2*	**10/12**	**15/15**
2	regulation of cell proliferation/GO:0042127	**10/1**	8/3	**21/24**	**23/31**
2	+ ve regulation of cell proliferation/GO:0008284	5/1	6/2	**14/13**	**13/17**
2	− ve regulation of cell proliferation/GO:0008285	*5/0*	*3/2*	**7/14**	**11/16**
3	regulation of cell growth/GO:0001558	4/1	3/2	**6/10**	**9/13**
3	regulation of cell cycle/GO:0051726	*2/1*	*1/4*	10/7	**8/15**
3	cell cycle checkpoint/GO:0000075		*0/2*	6/0	*4/3*
3	regulation of S phase/GO:0033261			*1/2*	**0/6**
3	cell cycle arrest/GO:0007050		*0/3*	*4/1*	**6/5**
3	cell division/M phase of cell cycle^a^/GO:0051301			*11/0*	**17/5**
3	cytoskeleton organization/GO:0007010			11/9	**9/16**
4	cellular response to stress/GO:0033554	*2/3*	*3/2*	16/6	**28/10**
4	response to DNA damage stimulus/GO:0006974			12/2	**21/6**
4	DNA damage response, signal transd./GO:0042770			4/2	**8/3**
4	response to oxidative stress/GO:0006979			6/4	7/6
4	homeostatic process/GO:0042592	7/2	6/6	**21/19**	17/22
5	− ve regulation of transcription/GO:0016481	*3/0*	*0/3*	**20/11**	**21/14**
5	+ ve regulation of transcription/GO:0045941	*5/0*	*2/4*	*11/9*	16/13
5	− ve regulation of nucleic acid metabol./GO:0045934	*3/0*	*2/2*	**24/11**	**23/17**
5	+ve regulation of nucleic acid metabol./GO:0045935	7/0	*4/4*	*13/11*	16/17
5	regulation of phosphorylation/GO:0042325	6/0	6/2	**17/14**	12/18
5	neuron differentiation/GO:0030182	*4/1*	*3/2*	**9/13**	11/13
6	+ ve regulation of cell communication/GO:0010647	4/1	*4/1*	**8/12**	**12/18**
6	regulation of synaptic transmission/GO:0050804	3/1	*4/1*	**4/7**	5/7
6	vesicle/GO:0031982	7/0	6/4	**17/13**	**17/22**
6	endocytosis^b^/GO:0006897	*3/0*	*3/0*	**8/5**	7/6
6	endomembrane system (ER, Golgi)/GO:0012505	*3/0*	*2/6*	21/7	*19/12*
7	vasculature development/GO:0001944	3/2	3/4	2/6	12/11
7	G_A_DB_DC/CARDIOVASCULAR	*9/1*	*9/2*	**20/24**	**18/31**
7	G_A_DB_D/diabetes, type 2	*3/0*	*3/3*	**11/13**	**15/14**
7	neuron development/GO:0048666	*4/1*	*3/2*	**7/12**	10/10
7	G_A_DB_DC/NEUROLOGICAL	*5/0*	*7/1*	9/16	11/26
7	G_A_DB_DC/AGING (Longevity)	*3/0*	*3/1*	**7/5**	**8/6**
7	G_A_DB_D/Alzheimer disease	*3/0*	*1/2*	5/11	**6/15**
7	G_A_DB_D/Parkinson disease			*3/4*	*4/6*
7	G_A_DB_DC/CANCER	*8/0*	*8/4*	*19/18*	23/25
7	G_A_DB_D/colorectal cancer	*2/0*	4/2	*6/6*	**7/12**

**Abbreviations: D:** disease; **GO:**Gene ontology; **G_A_DB_DC:** GENETIC ASSOCIATION DataBase _Disease_Class; **Notes: a:** GO:0000087; **b:** includes KEGG_PATHWAY hsa04144∼endocytosis; **c:** n = 75 for 1w5 nM, n = 122 for 1w50 nM, n = 417 for 4w5 nM and n = 619 for 4w50 nM; GO terms significant overrepresentation is indicated as follows: **P<0.005 by** **bold numbers**, P<0.05 by non-bold numbers, and *P>0.05 by numbers in italics*.

**Figure 2:**
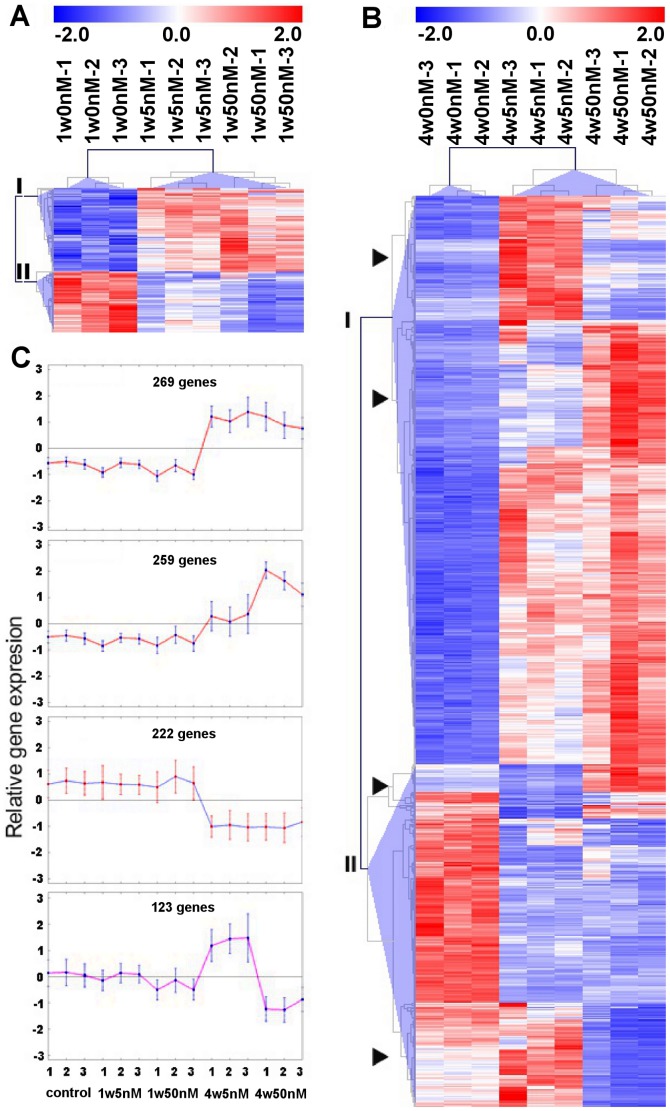
Hierarchical cluster analyses of microarray expression data. Overall cluster analysis of three transcriptome analysis experiments of SK-N-MC cells chronically treated with 5 nM, 50 nM rotenone, or vehicle (0 nM) for 1 week (1 w) and 4 weeks (4 w). Genes significantly altered in the treatment groups were clustered by hierarchical average-linkage analysis and shown in colorgrams depicting the expression level of the genes (rows) in each individual sample (columns). Expression above the mean is displayed in red and below the mean in blue (see normalized scale bar on top). (**A**) genes significantly altered by 5 nM and 50 nM rotenone at 1 week; (**B**) genes significantly altered by 5 nM and 50 nM rotenone after 4 weeks. Major cluster are indicated roman numerals and subclusters of genes that show opposite expression pattern at 4 weeks are indicated with arrowheads. The profiles in (**C**) depict the effects of both rotenone doses across both time points on the relative gene expression in four gene major clusters distinguished by the specific expression change exerted by each dose; the treatment group are indicated at the bottom and the number of changed genes are indicated at the top of each profiles.

The number of gene transcripts changed at least 2-fold by both doses at 1 week was a combined total of 134, of which 40% were commonly affected. The expression pattern of these genes is depicted as a heat map in **Fig. 2A**; where, clusters of genes induced or repressed by either or both doses are indicated by (I) or (II), respectively. Both doses induced the expression of 45 genes in common at 1 week, while the repression was more robust with the higher dose. At 4 weeks both doses altered the expression of a total of 825 genes, of which 30% were mutually affected. Their expression pattern is shown as a heat map in **Fig. 2B**, identifying two clusters (I and II) of altered genes and a few subclusters of genes affected differently by both doses (arrow heads). Four major types of changes caused by both doses across both time points are profiled in **Fig. 2C**. The results indicate that both doses exerted comparable and opposing cumulative effects on the expression of certain genes sets by a time-dose-dependent mechanisms.

### Validation of Microarray Results

**Figure 3:**
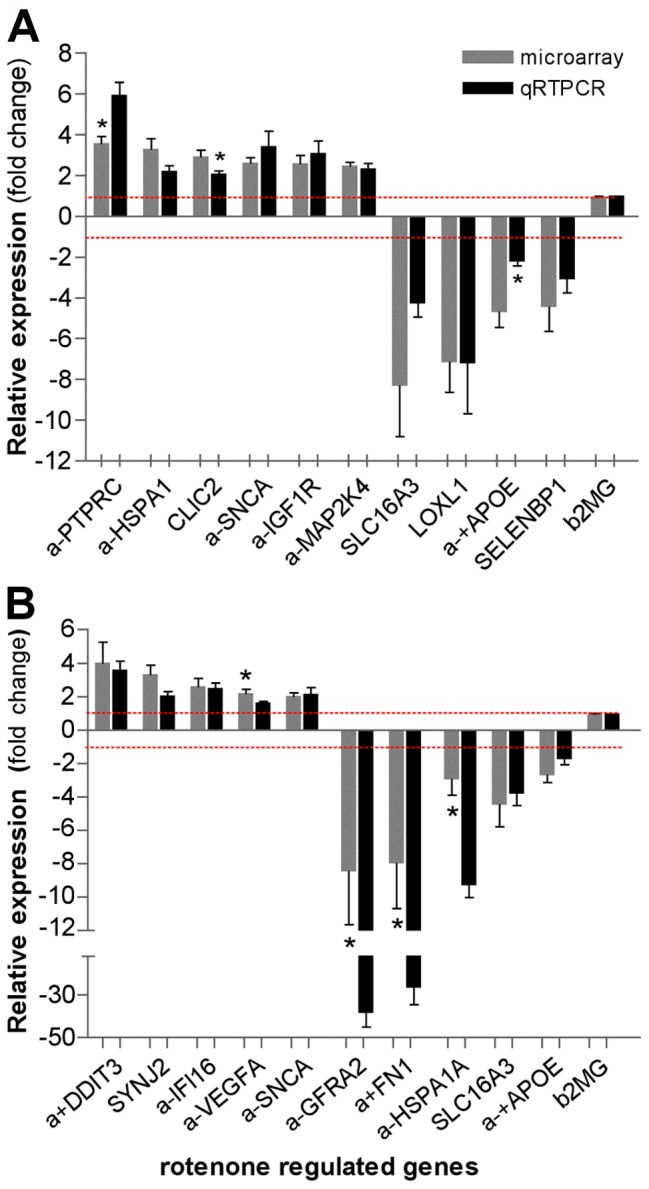
Validation of microarray results for differentially regulated genes (DRGs). The confirmation of selected differentially regulated genes was achieved by quantitative real-time polymerase chain reaction (qRT-PCR) using total RNA from SK-N-MC cells: (**A**) treated with 5 nM rotenone for 4 weeks and 0 nM (vehicle-treated control); (**B**) treated with 50 nM rotenone for 4 weeks and 0 nM (control). Comparison of the qRT-PCR analysis results of rotenone-treated samples to vehicle-treated samples showed that all genes changes were significantly different (P<0.05). The qRT-PCR average fold changes of selected DRGs in the rotenone treated samples, normalized to *B2M* (beta-2-microglobulin) and relative to expression on the vehicle-treated cells (dotted red line) are shown (mean ± SEM; *P<0.05; t-test, qRT-PCR vs microarray result, n = 3). Pearson’s test found significant correlation between microarray and qRT-PCR data in the 4w5 nM group (r^2^ = 0.9029, P<0.0001) and in the 4w50 nM group (r^2^ = 0.726, P<0.0017). *PTPRC* (protein tyrosine phosphatase, receptor type, C), *HSPA1A* (heat shock 70 kDa protein 1A), *CLIC2* (Chloride intracellular channel 2), *SNCA* (α-synuclein), *IGFR1* (IGF1 receptor), *MAP2K4* (mitogen-activated protein kinase kinase 4 ), *SLC16A3* (solute carrier family 16, member 3 -monocarboxylic acid transporter), *LOXL1* (lysyl oxidase-like 1), *APOE* (Apolipoprotein E), *SELENBP1* (selenium binding protein 1), *DDIT3* (DNA-damage-inducible transcript 3), *SYNJ2* (synaptojanin 2), IFI16 (interferon, gamma-inducible protein 16), *VEGFA* (vascular endothelial growth factor), *GFRA2* (GDNF family receptor alpha 2), and *FN1* (Fibronectin 1).

Microarray data was corroborated by qRT-PCR analysis of 10 selected genes from each treatment group at 4 weeks. The qRT-PCR data showed that all changes were significantly different (P<0.05) to controls in their respective cohorts, using the *B2M* mRNA as internal control. While comparison of the microarray with the qRT-PCR data showed that most selected genes in the 5 nM group (**Fig. 3A**) were changed in the same direction and not significantly different (P<0.05), except for the *APOE*, *CLIC2* and *PTPRC* genes. The direction of expression of all analyzed genes in the 50 nM group was the same (**Fig. 3B**); and the magnitude was similar with both methods for most genes; except for *VEGF,* which was slightly higher by qRT-PCR, and *GFRA2*, *FN1*, and *HSPA1A*, which varied widely by both methods. Yet, both methods coincided often, especially in the direction of the changes. Overall, the qRT-PCR analysis validated the microarray results; as indicated by the Pearson’s test which found significant correlation between microarray and qRT-PCR data in the 5 nM group (r^2^ = 0.9029, P<0.0001) and in the 50 nM group (r^2^ = 0.726, P<0.0017). Thus, gene expression changes were by and large accurately assessed by microarray analysis.

### Rotenone Affects Multiple Cellular Processes Involved in Neurodegeneration

Functional pathways affected by rotenone were identified by enrichment analysis using DAVID tools [36], as previously described [37]. Various sets of DRGs from the rotenone-treated groups were significantly (P<0.05) enriched in multiple “GO terms” (hereafter referred to as functional categories), which suggest multiple physiological alterations by rotenone. Thirty representative functional categories significantly enriched in at least 1 time point by either dose were further evaluated for their relevance to neurodegeneration and were organized into seven *ad hoc* function-related groups, with considerable overlap (**Table 1**). All these groups are intricately connected to cell fate through the regulation of apoptosis, cell cycle, proliferation, DDR, transcription and differentiation [38]–[40]. Dose-time dependence of rotenone effects on some of these processes has been seen in cell lines and primary neurons [7], [11], [17]–[19], [21], [27], [28], [41], [42]; but the underlying molecular mechanisms remain unclear. Mechanistic aspects relevant to NDs, as inferred from the functions of genes in the first six groups, are described below. The seventh group in **Table 1** comprises neuronal and vasculature tissues development processes and a number of human diseases that are associated with altered genes, according to the Genetic Association Database (GAD) of human disease; such genes are listed in **Table S3**.

### Apoptosis is a Prominent Feature of Neuroblastic Cell Fate Regulation by Rotenone

**Figure 4:**
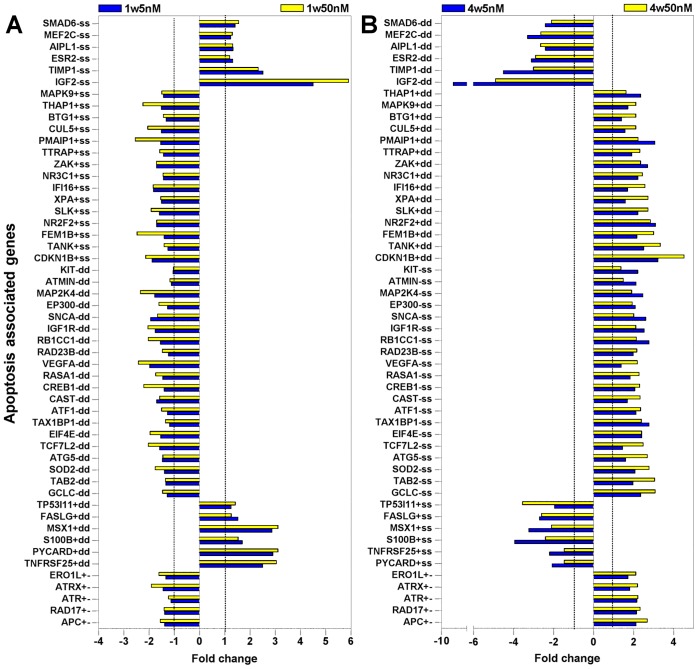
Expression patterns of apoptosis genes mutually altered by both rotenone doses. The expression levels of 52 apoptosis associated genes that were mutually and mostly similarly changed by both doses at 4 weeks are shown as fold changes relative to vehicle-treated cells. The expression patterns observed at 1 week (**A**) and at 4 weeks (**B**) of rotenone exposure are shown. The classification of each gene, according to their effect on apoptosis, as anti-apoptotic (−), pro-apoptotic (+), and ambivalent (+−) is indicated immediately after the listed symbol of each gene; followed by a further classification, based on the detected genes changes, into pro-survival events (indicated by s, to designate upregulation of anti-apoptotic genes and the downregulation of pro-apoptotic genes) and pro-death events (indicated by d, to designate upregulation of pro-apoptotic and the downregulation anti-apoptotic genes). Genes differentially changed by both doses are classified as both *s* and *d*; and ambivalent genes (+−) cannot be classified by these parameters. The 1-fold change level is indicated by dotted line.

**Figure 5:**
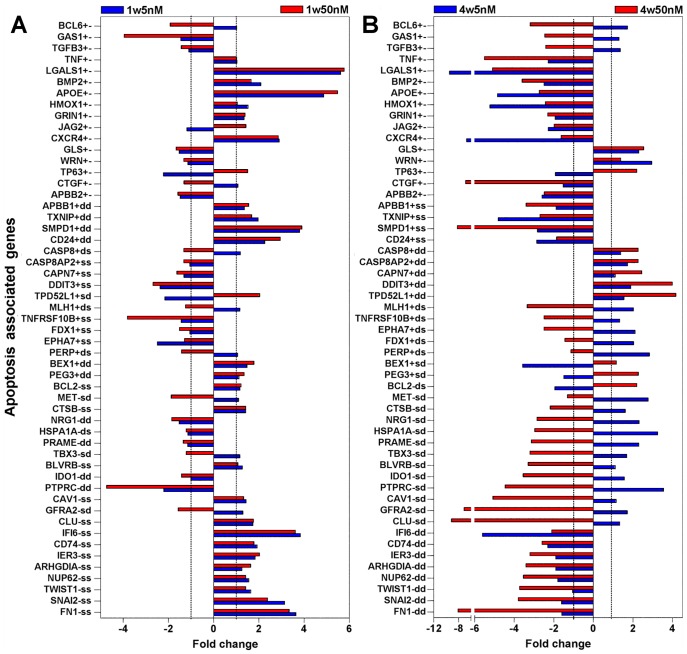
Expression patterns of apoptosis genes independently altered by either rotenone doses. The expression levels of 53 apoptosis associated genes that were changed mostly independently by either dose at 4 weeks are shown as fold changes relative to vehicle-treated cells. The expression patterns observed after 1 week (**A**) and after 4 weeks (**B**) of rotenone exposure are shown. The classification of each gene, according to their effect on apoptosis, as anti-apoptotic (−), pro-apoptotic (+), and ambivalent (+−) is indicated immediately after the listed symbol of each gene; followed by a further classification, based on the detected genes changes, into pro-survival events (indicated by s, to designate upregulation of anti-apoptotic genes and the downregulation of pro-apoptotic genes) and pro-death events (indicated by d, to designate upregulation of pro-apoptotic and the downregulation anti-apoptotic genes); ambivalent genes (+−) cannot be classified by these parameters. The 1-fold change level is indicated by dotted line.

**Figure 6:**
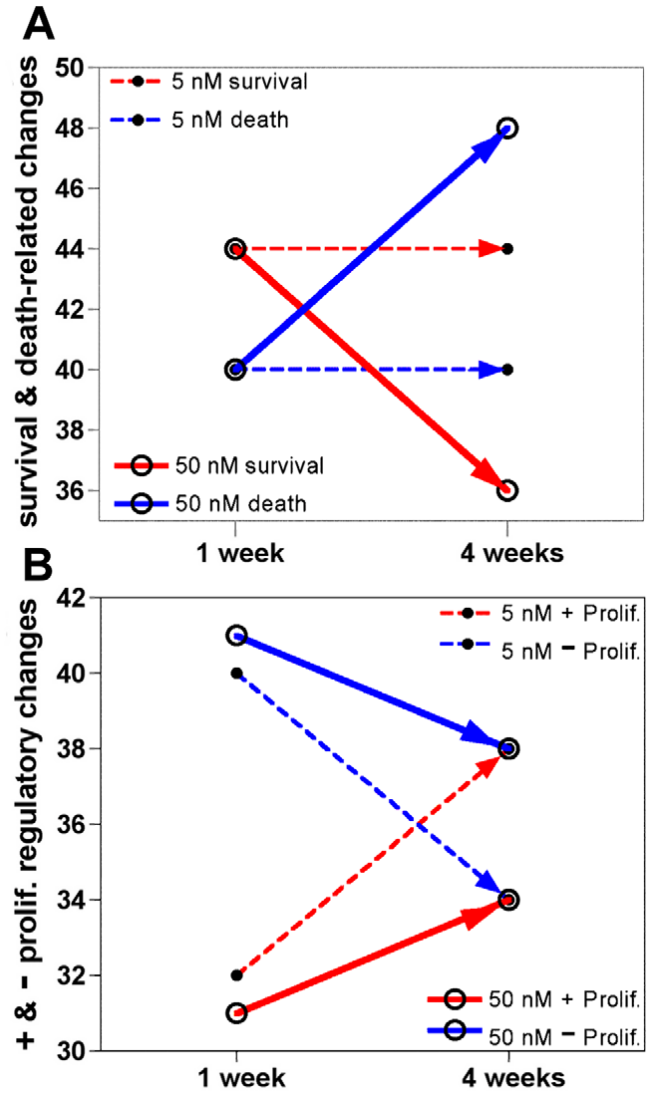
Streamlined illustration of the effects of rotenone on apoptosis and proliferation across time. (**A**) Rotenone-induced expression changes in apoptosis-associated genes, classified as pro-survival or pro-death events as defined in **Figs. 4** **&** **5**, at 1 week and 4 weeks were counted and plotted to reductively illustrate apoptotic differences between doses across time. (**B**) Rotenone-induced expression changes in cell proliferation-associated genes, classified as stimulatory (+) or inhibitory (−) events based on the assumption that increased negative regulators and decreased positive regulators of proliferation are inhibitory events and conversely, increased positive regulators and decreased negative regulators are stimulatory events were tallied and plotted to reductively illustrate proliferation difference between doses across time.

Apoptosis was the process most noticeably affected by rotenone at both doses. Given that apoptosis is a hallmark of neurodegeneration; this group was further analyzed to unveil apoptotic mechanisms regulated by rotenone at both doses. To this end, the initial DAVID-derived list of 63 rotenone-changed apoptosis genes was expanded by data mining to 105 genes; which is above 12% of all rotenone altered genes and represent apoptotic signatures of rotenone toxicity at such doses. The expression patterns of these genes (**Figs. 4** **&** **5**) depict trends in similarity and difference across time and doses, including magnitude and regularity of the changes. Genes whose expression was mutually and coordinately changed in the opposite way to changes at 1 week by both doses at 4 weeks are shown in **Figs. 4A & 4B**; and those changed, mostly independently, in a less synchronized way by either dose at 4 weeks are shown in **Figs. 5A & 5B**. As apoptosis proteins are restocked via transcription, the regulation of their gene expression may impact the balance of cell survival and death; thus allowing the use of their expression data to surmise survivability. Hence, the apoptotic genes were classified as anti-apoptotic (−), pro-apoptotic (+), and ambivalent (+−) regulators. Also, functionally, increased expression of anti-apoptotic genes and decreased expression of pro-apoptotic genes support cell survival (s); whereas increased expression of pro-apoptotic genes and decreased expression of anti-apoptotic genes favor cell death (d). A plot of these changes (**Fig. 6A**) revealed that at 1 week both doses promoted similar number of death and survival events. In contrast, the prevailing death-promoting capacity of the higher dose is obvious at 4 weeks; manifested as an increase in pro-death events and decrease in pro-survival events, while the lower dose apoptotic changes were virtually the same at 4 weeks. The tipping of the scale towards death by the higher dose and longer exposure is consistent with the dose-time dependence seen by others in NB cells, primary neurons and other cell types [5], [7], [11], [12], [17], [18], [27], [28], [41], [42]. Though the mutually affected apoptosis genes (**Fig. 4**) and the independently affected genes changed in similar direction (**Fig. 5**) by both doses suggest that, at low doses, rotenone could set up cells for apoptosis and make them more vulnerable to other insults, which may underlie the reported higher H_2_O_2_ susceptibility of cells chronically exposed to low rotenone amounts [11]. The upregulation of the expression of indicators of stress-related organelle dysfunction, such as *DDIT3*, *CAPN7*, *CAST* and *IFI16,* (**Fig. 5B**), suggests intrinsic apoptosis activation [14], [23], [43], [44]. While, extrinsic apoptosis activation is indicated by the induction of *CASP8* and *CASP8AP2* mRNAs [45], (**Fig. 5B**); and is consistent with previous observations [**42**]. Noteworthy, although the response to rotenone may involve the classical apoptosis pathways, it does not preclude alternative cell death mechanisms; particularly other cell cycle-linked cell death pathways [46]; since, as described below, rotenone seems to affect cell cycle progression in various ways.

### Rotenone Inhibits Cell Proliferation through Transcription Regulation

The majority of the affected cell proliferation genes (**Table 1**) also regulate apoptosis, the cell cycle and the DDR (**Figs. 4** **&** **5**, **Tables 2** & **3**). Such cross-utilization of proteins in opposing processes provides common homeostatic regulatory pathways to antagonistic processes [38]. Expression of most (72%) of the proliferation regulator genes was similarly affected by both doses, except that the magnitude of a number of changes induced by the 5 nM dose was below 2-fold. Functionally, increased downregulators and decreased upregulators of proliferation are inhibitory events; and, increased upregulators and decreased downregulators are stimulatory events. A tally of such events, as depicted in **Fig. 6B**, revealed that at 1 week the balance was tipped towards inhibition by both doses; albeit slightly more by the higher dose. At 4 weeks, the balance was tilted in favor of proliferation by the lower dose; whereas the higher dose still favored inhibition. These transcriptional events are, by and large, consistent with the proliferation analysis results described above (**Fig. 1**).

**Table 2:** Cell cycle progression-associated genes regulated by rotenone.

entrez	gene	fold c. (1week)		fold c. (4 weeks)		phase transition	references
ID	symbol	5 nM	50 nM	5 nM	50 nM	(function or effect)	
1027	CDKN1B	−1.9	−**2.1**	***+3.2***	***+4.6***	G0/G1/S (pro-senesc.)	[49], [120], [121]
596	BCL2	+1.0	*+1.3*	−1.7	***+2.2***	G0/G1/S/G2 (pro-senesc.)	[48]–[50]
8626	TP63	+1.0	*+1.1*	−1.9	***+2.2***	G1/S (pro-senesc.)	[122]
3398	ID2	+1.3	−*1.4*	+1.3	***−2.6***	G0/G1 (anti-quiesc.)	[51], [52], [123]
5925	RB1	***−***1.7	**- 3.4**	*+1.5*	*+1.6*	G0/G1/S/G2 (delays, arrest)	[74], [75]
5934	RBL2	***−***1.0	***−***1.1	*+1.9*	***+2.2***	G0/G1/G2(pro-senesc.)	[74], [75], [124]
6926	TBX3	+1.1	***−*** *1.2*	+1.7	***−3.2***	G1/S (anti-senesc.)	[53]
604	BCL6	+1.0	***−*** *1.9*	+1.7	***−2.8***	anti-senesc.	[54]
1191	CLU	+1.8	+1.8	+1.3	***−9.1***	anti-senesc.	[55]
595	CCND1	+1.5	+1.6	***−*** *1.4*	***−2.0***	G1/S (accel.)	[74], [125]
1869	E2F1	+1.5	+1.7	***−*** *1. 8*	***−*** *1.3*	G1/S/G2 (accel., senesc.)	[74], [75]
1870	E2F2	***−***1.0	***−***1.0	***−*** *1.4*	***−2.0***	G1/S/G2 (accel., senesc.)	[74], [75]
901	CCNG2	***−***1.3	***−*** **2.0**	***+2.1***	*+1.4*	S (replication/accel. )	[126]
4174	MCM4	+1.4	+1.4	***−*** *1.4*	***−2.4***	S (replication/accel. )	[127], [128]
990	CDC6	+1.5	+1.1	***−*** *1.4*	***−*** *1.9*	S (replication/accel. )	[127], [128]
9401	RECQL4	+1.7	**+2.0**	***−2.1***	***−3.7***	G1/S (accel.)	[129]
898	CCNE1	**+2.1**	+1.9	***−*** *1.4*	***−*** *1.5*	G1/S (replication,arrest)	[74], [125]
3084	CCNA1	+1.9	+1.7	***−*** *1.8*	***−2.9***	G1/S (replication, arrest)	[130]
6285	S100B	*+1.7*	*+1.5*	***−*** **4.0**	***−*** **2.4**	G1/S (arrest)	[131], [132]
6282	S100A11	*+1.8*	*+1.9*	***−*** **5.6**	***−***1.5	S/G2 (G2/accel.)	[131], [133]
1620	DBC1	*+1.1*	***−***1.1	***+*** *1.3*	***−*** **2.4**	G2/M (G2 arrest)	[56]
51512	GTSE1	+1.2	+1.3	***−*** *1.6*	***−2.4***	G1/S/G2 (arrest)	[134]
7164	TPD52L1	***−*** **2.2**	***−*** **2.0**	*+1.5*	***+4.2***	G2/M (MTdynamics)	[135]
8555	CDC14B	***−***1.2	***−***1.1	***+2.6***	*+1.2*	M (MT, accel. )	[136], [137], [138]
7465	WEE1	***−***1.1	***−***1.2	*+1.6*	*+1.9*	G2/M (accel. entry to M)	[136]
993	CDC25A	+1.4	+1.7	***−*** *1.9*	***−*** *1.5*	S/G2/M (accel. )	[139]
9748	SLK	***−*** *1.6*	***−*** *1.9*	**+2.2**	**+2.7**	S/G2/M (G2 arrest)	[140]
900	CCNG1	***−*** *1.4*	***−*** *1.6*	+1.3	**+2.4**	G2 (G2 arrest)	[60], [141]
891	CCNB1	***−*** *1.1*	***−*** *1.2*	+1.4	***−*** *1.8*	G2/M accel., M entry)	[142], [143], [144]
890	CCNA2	+1.0	***−*** *1.2*	+1.4	+1.5	G2/M (accel., MT distab)	[145]
49855	SCAPER	***−***1.8	***−***1.7	***+3.3***	***+4.0***	G2/M (accel., MT stab)	[145]
1647	GADD45A	***−***1.5	***−***1.3	***−***1.5	***+2.9***	G2/M (accel., MT stab)	[143]
9133	CCNB2	+1.2	+1.4	***−*** *1.2*	***−*** *1.3*	M (MT stabilization)	[142], [143]
827	CAPN6	+1.4	+1.4	***−3.0***	***−3.0***	M/I (arrest, MT stab, SAC)	[146]
10381	TUBB3	+1.4	+1.5	***−*** *1.4*	***−2.3***	M (accel., MT stab)	[61]
84617	TUBB6	**+2.0**	+1.7	***−*** *1.2*	***−2.7***	M (mitotic progression)	[62]
10382	TUBB4A	+1.6	+1.5	***−2.3***	***−3.6***	M (arrest, SAC)	[61]
9212	AURKB	+1.2	+1.3	***−*** *1.2*	***−2.0***	M (MT stab, Chr dynamics)	[147]
324	APC	***−***1.4	***−***1.6	***+2.1***	***+2.7***	G0/G1/S (pro-senesc.)	[63], [148]
6787	NEK4	***−***1.1	***−***1.2	*+1.7*	***+2.6***	G0/G1/S/G2 (pro-senesc.)	[149]
91754	NEK9	***−*** *1.1*	***−*** *1.3*	**+2.1**	***−2.6***	G1/S (pro-senesc.)	[147], [150]
55743	CHFR	*+1.4*	*+1.3*	*+1.1*	***−*** **2.3**	G0/G1 (anti-quiesc.)	[63], [64], [72]
110076	TPPP	+1.3	+1.4	***−4.2***	***−4.7***	G0/G1/S (anti-quiesc.)	[65]

**Abbreviations**: Accel.: accelerates; fold c.: fold change; M: mitosis; MT: microtubules; SAC: spindle assembly checkpoint; senesc: senescence; stab: stabilizes; destab: destabilizes. Changes likely to delay the cell cycle are shown by *numbers in italics*; changes likely to accelerate cell cycle are shown by regular numbers; emboldening indicates fold change is <2.

**Table 3:** DNA damage response-associated genes regulated by rotenone.

entrez	gene	fold c. (1 week)		fold c. (4 weeks)		repair mechanism	references
ID	symbol	5 nM	50 nM	5 nM	50 nM	(function)	
545	ATR	***−***1.1	***−***1.2	***+2.2***	***+2.2***	SSB, DSB (sensor, activator)	[39], [40]
1111	CHEK1	***−***1.3	***−***1.7	***+2.0***	*+1.3*	SSB, DSB (sensor, effector)	[39], [40]
5884	RAD17	***−***1.4	***−***1.4	***+2.2***	***+2.3***	SSB, DSB, BER, HR (sensor)	[39], [40]
472	ATM	***−***1.1	***−***1.1	*+1.8*	*+1.5*	DSB, (sensor, activator)	[39], [40]
11200	CHEK2	*+1.2*	*+1.2*	+1.0	+1.0	DSB, (sensor, effector)	[39], [40]
4361	MRE11A	***−***1.6	***−***1.7	***+2.0***	*+1.9*	DSB, HR (sensor, mediator)	[39], [40], [68]
10111	RAD50	***−***1.3	***−***1.3	*+1.6*	*+1.4*	DSB, HR (sensor, mediator)	[39], [40], [68]
4683	NBN	+1.0	***−***1.2	*+1.6*	*+1.4*	DSB, HR (sensor, mediator)	[39], [40], [68]
51776	ZAK	−1.7	−1.7	***+2.7***	***+2.4***	DSB, (activator, arrest)	[151]
25788	RAD54B	+1.0	−1.3	***+2.0***	***+2.2***	DSB, HR (helicase)	[152]
5980	REV3L	−1.1	−1.2	***+2.2***	***+4.2***	DSB, TLS, HR (polymerase)	[153]
7486	WRN	−1.1	−1.3	***+3.0***	*+1.4*	DSB, HR, TLS (helicase)	[154]
51567	TDP2	−1.4	−1.6	*+1.9*	***+2.3***	DSB,NHEJ (phosphatase)	[68], [155]
4750	NEK1	−1.5	−1.5	***+2.4***	***+3.2***	DSB, HR, TLS, MMR (kinase)	[156], [157]
5887	RAD23B	−1.2	−1.5	***+2.0***	***+2.2***	NER (proteasome)	[68]
1161	ERCC8	−1.2	−1.4	*+1.5*	***+2.0***	NER (proteasome)	[68], [158]
7507	XPA	−1.5	−1.6	*+1.6*	***+2.7***	NER (DNA binding)	[68], [158]
4436	MSH2	−1.0	−1.3	*+1.2*	*+1.1*	MMR (sensor)	[67], [69], [70]
2956	MSH6	−1.2	−1.3	*+1.5*	*+1.2*	MMR (sensor)	[67], [69], [70]
4437	MSH3	−1.3	−1.5	−1.1	*+1.1*	MMR (sensor)	[67], [69], [70]
4992	MLH1	*+1.2*	−1.2	***+2.0***	−**3.3**	MMR, HR (sensor, repair)	[67], [69], [70]
5395	PMS2	*+1.4*	*+1.4*	−1.1	−1.1	MMR, HR (repair)	[67], [69], [70]
5378	PMS1	−1.3	−1.3	*+1.4*	***+2.2***	MMR, HR (repair)	[67], [69], [70]
8930	MBD4	*+1.2*	−1.1	*+1.7*	−1.5	MMR, HR (sensor, repair)	[159]

**Abbreviations: BER:** Base excision repair; **DSB:** DNA double strand break; **HR:** Homologous recombination; **MMR:** Mismatch repair; **NHEJ: NHEJ**: Non-homologous end-joining; **NER:** Nucleotide excision repair**; SSB:** DNA single strand break; **TLS:** Translesion. Changes likely to delay cell cycle are shown by *numbers in italics*; those likely to accelerate cell cycle are shown regular numbers; **Emboldening** indicates fold change (fold c.) is <2.

### Rotenone Affects Cell Cycle Progression through Interphase

The majority (73%) of the cell cycle regulator genes, whose expression was changed above 2-fold by either dose, was similarly affected; except that the magnitude of most changes was below 2-fold at 1 week and a few in the 5 nM-treated cells at 4 weeks. To ascertain links between expression levels and inclination of treated-cells to maintain or delay cell cycle progression, the function of each regulated gene was evaluated. The genes were then ordered based on their functions as shown in **Table 2**; including a few genes whose change was below 2-fold as phase markers. Due to the number of cell cycle genes affected by rotenone, the functions of each of these genes is not discussed here; instead, references for the pertinent functions of each gene are listed in **Table 2**. The data reveals that at 1 week cells exposed to both rotenone doses could traverse the cell cycle with likely delays at the routine cell cycle checkpoints. In contrast, at 4 weeks, both doses may cause delays or arrest at various checkpoints; with a more prominent effect by the higher dose. Some of the DRGs encode key regulators of the cell cycle transitions and checkpoints and could be responsible for cell cycle delays. Indeed, such expression changes suggest that some cells treated with the 50 nM dose for 4 weeks express a senescence-like phenotype or may have differentiated into N-type phenotype. As the high expression of *BCL2* and *CDKN1B* in cells treated with the higher dose (**Table 2**) has been associated with differentiating, quiescent and senescent NB cells [47]–[50]. Conceivably, as described later, rotenone may promote differentiation; and such a notion is supported by the markedly reduced expression, by the higher dose, of the gene for ID2, a protein required for cells to exit G0 that is repressed in senescent cells [51], [52]. More support comes from the repression of genes whose proteins prevent senescence namely *TBX3* [53], *BCL6* [54], and *CLU* [55]. Together these results suggest that the higher dose and the lower dose, to a lesser extent, may induce cell cycle arrest, which could lead to differentiation, quiescence, senescence and apoptosis. Noteworthy, repression by the higher dose, of G1/S transition inhibitors like *DBC1*, a gene often suppressed by methylation [56], suggests that cells with damaged DNA may bypass checkpoints via epigenetic silencing; which is backed by the effects of rotenone on various epigenetic pathways, described later.

It should be noted that the effects of rotenone on the cell cycle of NB cells are likely to be connected more to their neoplastic lineage than to the phenotype of post-mitotic neurons that do not divide. Nevertheless, the expression of many cell cycle regulators is known to continue in adult neurons (see review [57]), which has led to suggestions of crucial cell cycle independent roles for such proteins [57], [58]. Also, the evidence suggest that the upregulation of cell cycle proteins in differentiated neurons under a variety of stress conditions, including OS and exposure to genotoxins, is part of a well orchestrated mechanism of cell death; distinct from the classic apoptosis pathways, which is triggered by abortive cell cycle attempts likely due to neuronal structural constraints to undergo mitosis [57]. However, our data shows that rotenone downregulates the cell cycle, which may become relevant to neuronal function if the affected genes have adopted non-canonical functions. For instance, CDK5, which is involved in synaptic plasticity and memory; and is active in adult neurons [57], was decreased ∼90% (not shown) by both doses. Moreover, as evidence suggest a link between synaptic plasticity changes and regulation of differentiation, cell cycle repression, and cell death in mature neurons [57], [58]; changes to cell cycle proteins that also impact apoptosis, differentiation, epigenetic pathways, and the MT-system components, as discussed elsewhere, could be detrimental to neurons.

### Rotenone Transcriptionally Impacts Microtubule Stability and Mitosis Progression

The numerous rotenone-deregulated genes encoding proteins that associate with or that are components of the cytoskeleton, in particular the MT system (**Tables 1** **&** **2**), suggest that rotenone disrupts mitosis by transcription regulation. As manifested by changes in the expression of genes for MT components and associated proteins, which alter MT dynamics and impact mitotic arrest; thus influencing processes involved in cell fate decisions leading to various outcomes, including, survival and continuing cycling often as polyploid, arrest and senescence, and cell death by various mechanisms including apoptosis [59], [60]. Such as, the downregulation, at 4 weeks, of the expression of the genes for the following proteins: TUBB3, TUBB4, and TUBB6, which are neuronal tubulin β isotypes, essential components of the MT, involved in MT dynamics and control of the spindle assembly checkpoint (SAC) that triggers mitotic arrest, which could lead to continuation of the cell cycle, chromosomal aberrations if slippage occurs, or apoptosis [59], [61], [62]. The upregulation of the gene for APC, which modulates MT dynamics and the SAC [63], also suggests that rotenone may trigger the SAC. Other interesting changes include the differential effect of rotenone on expression of the *CHFR* gene, and the suppression of the *TPPP* and *CAV1*genes. CHFR controls a mitotic stress checkpoint in response to MT destabilization and maintenance of chromosomal stability [63], [64]. Thus, its suppression may allow MT-defective cells to bypass the CHFR-checkpoint and promote genomic instability. The tubulin polymerization promoting protein (TPPP), whose mRNA expression was repressed (>4 fold, see **Tables S2C & S2D**) by rotenone, is also involved in MT stabilization [65]. The downregulated *CAV1* gene (>5 fold, see **Tables S2D**) encodes for another upregulator of MT polymerization [66]. Collectively, these expression changes strongly support the notion that, besides its direct MT destabilizing property [27], rotenone affects MT system stability at the transcriptional level and hence can impact essential MT-associated processes, including cell division, genomic stability, axonal transport, OS and apoptosis. Such regulatory step could be the result of a feedback mechanism activated by the direct MT-depolymerization effect of rotenone [24], [27] and could contribute to the complex I-independent toxic effects of rotenone; which has been convincingly demonstrated by Choi et al., both in vivo and in vitro in complex I-deficient and wild type dopaminergic neurons [17], [18]}; though complex I inactivation strengthened rotenone toxicity [18].

### Rotenone-induced Cellular Response to Stress Involves DNA Damage Responses

**Figure 7:**
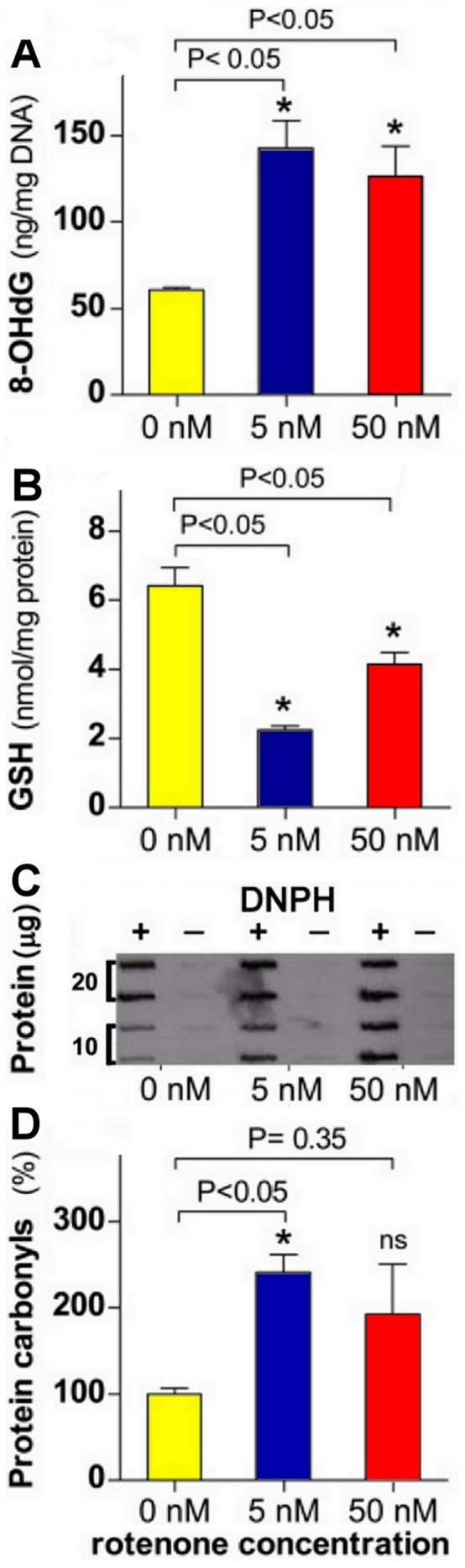
Chronic exposure to rotenone increases cellular oxidative stress. SK-N-MC cells were cultured in the absence or presence of a marginally toxic (5 nM) or a moderately toxic dose (50 nM) of rotenone for 4 weeks. Results are shown as the mean ± SEM, determined for each treatment group in 3 independent experiments measuring the levels of the following oxidative stress markers in cells treated with vehicle (0 nM), 5 nM and 50 nM rotenone: (**A)** Cellular levels of the DNA adduct 8-OH-dG (8-hydroxy-deoxyguanosine); results are shown in ng/mg of DNA. (**B)** Total cellular glutathione (GSH) levels; results are shown in nmol GSH/mg of protein. (**C)** Representative dot-blot of cellular protein carbonyls levels in controls and cells treated with 5 nM and 50 nM rotenone, used to assess protein carbonyls levels in D, using 10 and 20 µg of protein in the presence or absence (background control) of 2,4-dinitrophenylhydrazine (DNPH) solution. (**D)** Cellular protein carbonyls levels in controls and rotenone-treated cells; the average percentage of protein carbonyls levels in the rotenone treated cells normalized to the vehicle-treated control is shown. Mann-Whitney t-test, treated vs. control *P<0.05, ns: not significantly different.

The cellular response to stress comprises interconnected pathways through which ongoing processes like those in the first three groups are orchestrated; such as, the response to OS and various components of the DDR network. The DDR network facilitates resolution of DNA replication problems and integrates them with processes like the cell cycle, transcription, senescence and apoptosis [39], [40]. DDR repair pathways were induced by rotenone at 4 weeks (**Table 3**); as indicated by the upregulation of the genes for mediators of the repair of double and single-strand breaks (DSB & SSB), mismatches, oxidized bases and adducts [39], [40], [67], [68]. The upregulation of the genes *ATR*, *CHEK1*, *RAD17*, *ATM* and *CHEK2*, whose proteins are crucial for DNA damage recognition during homologous recombination (HR), non-homologous end-joining (NHEJ) and nucleotide excision repair (NER) [39], [40], [67], suggest that both ATR/CHEK1 and ATM/CHEK2 pathways were activated by both rotenone doses (**Table 3**). A view supported by the upregulation of the genes *MRE11*, *RAD50*, and *NBN* of the MRN complex that triggers the ATM/CHEK2 pathway [39], [68]. The mismatch repair (MMR) network, comprising the essential proteins MSH2, MLH1 and PMS2 [69], [70], repairs post-replication mismatches and triggers cell cycle arrest and apoptosis following DNA damage by ROS [67], [69], [70]. Thus, the dose-dependent repression of the gene for MLH1 (**Table 3**) raises the possibility of epigenetic expression silencing which would make cells MMR-deficient and hence more tolerant to rotenone’s DNA-damaging action. Reminiscent of tolerance mechanisms seen in cells exposed to ROS and DNA damaging agents that generate adducts such as 8-hydroxy-deoxyguanosine (8-OH-dG) [67], [70], which was increased by rotenone (**Fig. 7A**). Notably though, DSBs, that arise from replication fork arrest and collapse, are mainly repaired by HR and NHEJ [39], [40], [68]; two mechanisms upregulated by rotenone in this study (**Table 3**); which suggest that ROS-independent mechanisms may underlie the induction of the DDR by rotenone. Similarly, the higher GSH levels (**Fig. 7B**), and slightly lower levels of two OS markers in cells treated with the higher dose (**Figs. 7A & 7C**), which may reflect adaptive responses; yet higher level of transcriptional apoptotic response (**Figs. 4** **,** **5**, & **6A**) and of cell death (**Fig. 1D**) with such dose, also suggest OS-independent pathways in the rotenone response. In support of an adaptive response, expression of various anti-OS-related genes was regulated by rotenone, including two that were robustly increased by the higher dose, whose proteins impact ubiquitous antioxidative mechanisms; *SOD2* (**Fig. 4B**), a known rotenone-induced gene [15], and *GCLC* (**Fig. 4B**), a component of the rate-limiting enzyme in GSH synthesis [**71**]. Such adaptive response may reflect, as described elsewhere, rotenone-induced differentiation into neuroblastic phenotype, which may make them less sensitive to OS.

Importantly, changes leading to MMR-deficiency may also be partly responsible for the association of rotenone with cancer (**Table 1**). Indeed, changes like the downregulation of *MLH1* and *CHFR*, often caused by epigenetic silencing, are common in colon tumors and have been linked to the cancer phenotype [63], [64], [70], [72]; and are consistent with findings showing that rotenone exposure enhances tumorigenesis [73].

### Epigenetic Regulatory Mechanisms May Mediate Rotenone Effects on Cell Fate

Transcription regulators control various processes, including transcriptional activation and epigenetic silencing, which coordinate the timely supply or removal of important mediators of ongoing cellular processes [74]–[80]. Many rotenone-regulated genes, including those listed in **Table 4**, are involved in epigenetic pathways that crosstalk to control neurogenic [78] and other pathways whose dysregulation is associated with aging-related diseases [77]. For instance RBL2 and SUV420H1 are key components of the DREAM complex [76] that represses genes involved in cell cycle, differentiation, and senescence [74]–[77], which is consistent with the rotenone effects detected here. Also, REST and RCOR3 are components of the REST complex, which silences repressor element 1-containing neuronal genes in neurons and non-neuronal cells and controls adult neurogenesis [78]. Thus, the upregulation of such genes may drive SK-N-MC cells into a quiescent- or senescent-like state as suggested above. Rotenone also induced the mRNAs of the HIRA and ASF1A proteins that form the HIRA/ASF1A complex, which is also involved in repressing pro-proliferation genes in senescent cells [80]. Furthermore, BMI1, whose gene was also upregulated at 4 weeks (**Table 4**), is a key component of the Polycomb Repressive Complex 1 (PRC1), a major epigenetic silencing complex involved in neurogenesis, senescence, and apoptosis [78], [81]. Also, increased *BMI1* expression has been detected during the differentiation of the I-type SK-N-MC cells into the N-type [34]. Thus, the upregulation of *BMI1* by rotenone may contribute to such conversion into N-type; a view supported by the expression pattern of the genes listed in **Table S4**, often used as phenotype markers for NB cells [31], [33], [47], [48], [82].

**Table 4:** Rotenone regulated genes associated with transcription/epigenetic silencing pathways.

entrez	gene	fold c. (1 week)		fold c. (4 weeks)		complex	references
ID	symbol	1w5 nM	1w50 nM	4w5 nM	4w50 nM	(epigenetic silencing)	
5934	RBL2	−1.0	−1.1	+1.9	**+2.2**	DREAM	[74]–[76]
5111	SUV420H1	−1.4	−1.3	+1.9	**+2.7**	DREAM	[77]
5933	RBL1	−1.2	−1.1	+1.3	+1.9	DREAM	[74], [76], [160]
5978	REST	−1.0	−1.3	**+2.2**	**+2.1**	REST	[161], [162].
55758	RCOR3	−1.3	−1.7	+1.9	**+2.0**	REST	[161], [162].
25842	ASF1A	−1.4	−1.7	+1.4	+1.8	HIRA/ASF1A	[80]
7290	HIRA	−1.2	+1.0	+1.4	**+2.1**	HIRA/ASF1A	[80]
55723	ASF1B	**+2.9**	**+2.9**	−**2.1**	−**2.4**	CHAF1A/ASF1B	[79]
10036	CHAF1A	+1.3	+1.4	−1.8	−2.4	CHAF1A/ASF1B	[79]
648	BMI1	−1.4	−1.7	**+2.1**	**+2.3**	PRC1	[163]
7291	TWIST1	+1.6	+1.4	−1.0	−3.7	PRC1	[164]

**Notes: emboldening** indicates fold change (fold c.) is <2.

### Effects of Rotenone on Signaling, Neurotransmission, Endomembranes

The marked effect of rotenone on cytoskeleton organization, endocytosis, vesicle and the endomembrane system (endoplasmic reticulum (ER) and Golgi complex) related genes (**Table 1**), may affect functions relevant to NDs. Such as, axonal transport and amyloid precursor protein (APP) processing [83]–[85]; and vesicular trafficking, which requires an intact cytoskeleton and thus is disrupted by rotenone through its direct MT-depolymerization action [18], [24]}, and possibly, as detected here, by dysregulation of cytoskeleton genes (described above) and vesicles-related genes such as SNCA [86]. Proper functioning of the ER is critical for neuronal cell function; as it is the main site for Ca2+ homeostasis, and protein synthesis, folding and processing [87]–[89]; and is involved in cholesterol homeostasis [85], [90], [91] and vesicle trafficking [92]. Not surprisingly, ER dysfunctions are associated with pathophysiological aspects of various NDs, including PD, AD and their variant [88], [89], [93]–[99]; such as, accumulation of misprocessed proteins like SNCA, tau (MAPT) and Aβ. Rotenone-induced ER-stress is indicated by the marked upregulation of *DDIT3* (**Fig. 5B**), and is consistent with reports of rotenone-induced ER-stress in, in vitro and in vivo, models of PD; with concomitant aggregation of tau, Aβ and SNCA [7], [11], [20], [94], [98]. Such rotenone-induced ER-stress can lead to cell death [43], [95]. A summary of the interplay of proteins from rotenone-induced genes linked to signaling cascades, apoptotic, and cytoprotective pathways is shown in **Fig. S1**.

### Comparison to Previous Transcriptome Analysis of the Response to Rotenone

A transcriptome analysis of SK-N-MC cells treated with 5 nM rotenone, seemingly similar to ours, was previously reported [29]. However, the results differ widely from ours; thus, a comparison of the two data was warranted to explain the discrepancies. The Greene et al., (2008), [29], dataset (GSE4773; NCBI GEO databank) was analyzed like ours using dCHIP [100]. Comparison of QC parameters and results from both datasets at 4 weeks revealed lower median intensity and percentage of present calls in their data than in ours (see **Table S1 & Table S5**). In addition, the percentage of DRGs overlap in both studies was just 7% (58 of 841 DRGs). However, such differences in QC cannot explain such low overlap; especially, as both studies were performed in the same cell line, in the same platform, at the same microarray core facility; which should yield ∼90% overlap [101]. The most prominent difference between the datasets is in the magnitude and direction of the changes; as illustrated by the expression patterns of the 58 common DRGs in both datasets (see **Fig. S2A & Fig. S2B**); which becomes obvious when depicted under the same scale (**Fig. S2C)**, showing the magnitude of the changes in their study was rather faint and in opposite direction to ours; and echoes their own observations of 30% increase in intensity at 4 weeks [29]. Such attenuated rotenone effects could be due to the use of 5 mM sodium pyruvate in their culture medium [29]; which may counteract some of the rotenone effects, and may explained the slightly stronger proliferation inhibitory and cytotoxic effects of the 5 nM dose in our study (**Fig. 1**). In support of this notion, pyruvate has been shown to prevent some of the cytotoxic effects of rotenone on NB cells [25], [26].

### Concluding Remarks

**Figure 8:**
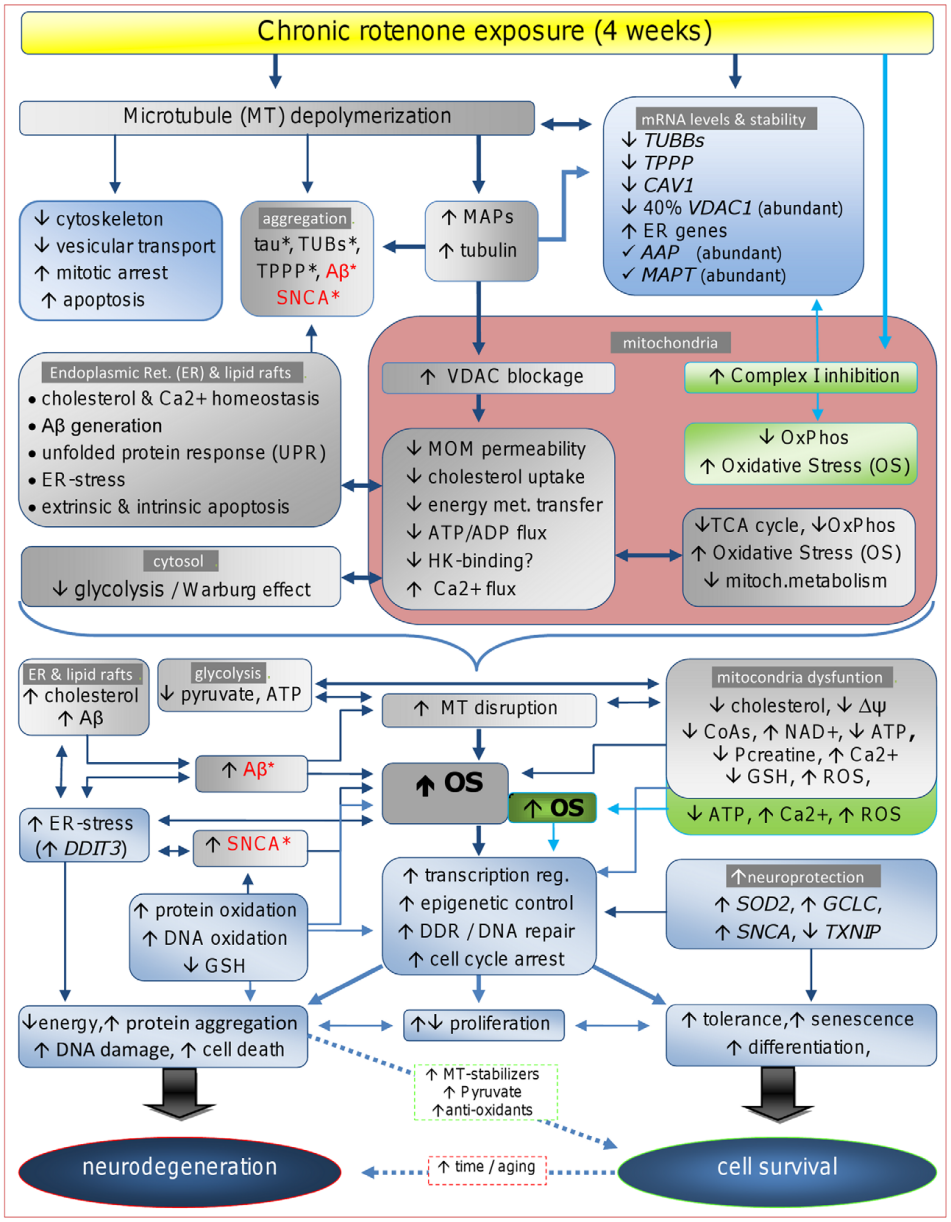
Proposed mechanisms. Schematic summary of mitochondria complex I-independent and dependent pathways affected by chronic exposure to rotenone; supported by our results and known cellular effects of rotenone-induced MT depolymerization. Complex I-dependent or independent effects are listed in boxes shaded in green or gray color, respectively. Cellular processes or components, and genes expression affected by rotenone in our study are listed in boxes shaded in blue color. Upward (↑) and downward (↓) arrows indicate up and downregulation, respectively. Detailed descriptions and interpretations on gene and pathway changes can be found in the results and discussion.

Our results, coupled with evidence of the direct effect of rotenone on MT stability [17], [18], [24], [27], [102], suggest plausible mechanisms for the response to rotenone, as summarized in **Fig. 8**, featuring the MT-depolymerization activity of rotenone in the triggering of ND-associated pathways independently of its complex I-inhibitory activity. Such that, MT disruption, likely partly sustained through rotenone-induced repression of *TPPP, CAV1,* and other MT-stabilizing genes, increases cytosolic tubulin; which, as detected in our study, triggers the degradation of its own mRNA [103]. Excessive cytosolic tubulin obstructs voltage-dependent anion channels (VDAC), which causes depolarization [102], decreases membrane potential (ΔΨm), and reduces the flux of superoxide ions, ATP/ADP and other mitochondrial metabolites. Such changes alter Ca2+ homeostasis, reduce OxPhos and ATP production, increase ROS generation and OS, and may trigger cell death pathways [104]–[107]. Also, VDAC blockage suppresses glycolysis [102], [104], [106] and thus pyruvate generation. Glycolysis suppression may be detrimental to cells with high energy demands such as neurons [108], and cancer cells, like SK-N-MC cells, as it contributes most of their cells energy demands [102]. Moreover, as the glycolytic phenotype is linked to high cholesterol uptake by mitochondria through VDACs after hexokinase II (HK) binding [109]; increased tubulin may displace HK from the VDAC [105] and reduce cholesterol uptake thus leading to its accumulation in the ER, which induces Aβ accumulation, and ER-stress. The interference of tubulin with HK-VDAC binding, to our knowledge, has not been reported; however, two published studies, together, suggest that rotenone indeed interferes with HK binding to VDAC, anti-apoptotic activity, and coupling of glycolysis to intramitochondrial OxPhos. First, phosphorylation of VDAC by glycogen synthase kinase-3b (GSK3B) was shown to be a crucial enhancer of VDAC-tubulin binding [110]. Secondly, chronic inhibition of GSK3B was shown to protect against rotenone-induced apoptosis, and led to enhanced glycolysis and accumulation of HK in the mitochondria; whereas GSK3B overexpression enhanced rotenone-induced cell death [111]. Also rotenone inhibits acetyl-CoA and succinyl-CoA generation during the Krebs cycle in NB cells [112] and pyruvate seems to attenuate the rotenone anti-proliferative and apoptotic response detected in our study [29]. Thus, it is likely that rotenone suppresses glycolysis and pyruvate production by tubulin blockage of VDAC. Other support for the posited pathways in **Fig. 8**, comes from findings, that rotenone enhances processing of APP into Aβ [7], [113], [114]; that MT disruption is linked to Aβ-induced elevated NAD+ levels, reduced ATP levels, and increased cell death [115]; and that PD cybrids with excessive tubulin had increased SNCA oligomer accumulation and lower ATP levels [116]. As such pathways do not require complex I inhibition to trigger the cascade of events leading to cell death; it is thus consistent with evidence indicating that rotenone-induced neuron cell death involves complex I-independent mechanisms that boosts its complex I inhibitory activity [17], [18]. These and other aspects of the response to rotenone uncovered in this study, including the upregulation of epigenetic regulatory mechanisms and the possible impairment of the MMR system warrant further investigation as possible therapeutic avenues not only for NDs but also for cancer.

## Materials and Methods

### Cell Lines and Culturing

An in vitro model of PD using the SK-N-MC human NB cell line [11] was used in this study with important modifications to the growth medium. Cells were maintained in a 5% CO_2_ environment at 37°C, in Eagle’s MEM medium with Earle’s salt (Invitrogen, Carlsbad, CA), supplemented with 5.6 mM D-glucose, 2 mM L-glutamine, non-essential amino acids, 50 U/ml penicillin and streptomycin, and 8% fetal bovine serum (Invitrogen). As pyruvate protects cells against some of the effects of rotenone [25], [26], it was not used during the course of the experiments. Cells were treated for 1 and 4 weeks with two different amounts of rotenone or vehicle-treated (0.05% ethanol). The 5 nM dose is, to some extent, marginally lethal (∼5% apoptosis, at 4 weeks) for SK-N-MC cells [11]; while the 50 nM dose causes more death (40–60%) of cultured SH-SY5Y NB cells [12], [21], [22].

### RNA Preparation, Microarray Processing and Data Analysis

Total RNA was extracted with Trizol™ (Invitrogen) from triplicate experiments from vehicle-treated and from 4 rotenone-treated groups: 1w0 nM and 4w0 nM (vehicle-treated for 1 week and 4 weeks), 1w5 nM and 4w5 nM (treated with 5 nM), 1w50 nM and 4w50 nM (treated with 50 nM). After QC analysis by the Agilent Bioanalyzer System (Agilent, Foster city, CA), the mRNA was used to generate the cRNA labeled probes used to hybridize to the human HG-U133A GeneChip® DNA array (Affymetrix), following the manufacturer’s protocol, at the UCLA microarray core facility (http://microarray.genetics.ucla.edu/). Data (GEO database accession # GSE35642) was then normalized and used to assess expression indexes and fold changes (FC >2.0, compared with vehicle-treated controls) using the model-based expression indexes (MBEI) method implemented in dCHIP, (http://biosun1.harvard.edu/complab/dchip/), [100]. Lists of differentially-regulated genes (DRGs) across samples were generated as described in the legend to **Table S2**, by filtering the data using dCHIP and by correcting for multiple testing by the Significance Analysis of Microarray method [117], implemented in the Multi-experiment Viewer (MeV) of the TM4 suite, (http://www.tm4.org), [118]. After corrections, the gene lists were reduced to 75, 112, 457 and 619 in the 1w5 nM, 1w50 nM, 4w5 nM and 4w50 nM groups respectively. Clustering analysis, using the average linkage method, was performed using the MeV, and enrichment analysis was performed using DAVID (http://david.abcc.ncifcrf.gov), to ascertain sets of rotenone DRGs enriched in certain biological annotations. Apoptosis genes in the list of DRGs were found by exhaustive literature searches and in the University of Michigan list of apoptosis regulators (http://www.personal.umich.edu/~/List/Alist.html).

### Real-time Quantitative PCR Validation of Results

Gene selection to validate microarray results was done primarily to encompass low, moderate and high intensity signal genes representative of the identified functional categories. Quantitative real-time RT-PCR (qRT-PCR) analysis was performed on RNA from a set of 10 selected genes from each treatment groups at 4 weeks. Total RNA (500 ng) was reverse transcribed using the Superscript III Kit (Invitrogen). Used primers (see **Table S6**) were custom made (Invitrogen). All PCR reactions were performed using a SYBR Green kit (Qiagen) and run in triplicate in the ABI 7700 System (Applied Biosystems Inc, Fullerton, CA). Transcripts were quantified by the comparative threshold cycle Ct method [119], comparing the target Ct values to Ct for the reference gene beta-2-microglobulin (*B2M*), thereby normalizing for small differences in starting template. All primer sets had PCR efficiencies comparable to the reference *B2M*, as ascertained by analysis of across serial dilutions of template (10-fold).

### Assaying Rotenone Effects on SK-N-MC Cells Proliferation and Cytotoxicity

SK-N-MC cells proliferation kinetics and cytotoxicity under rotenone exposure was ascertained as described in the legend to the proliferation curves in **Fig. S3**. The data from such curves under each treatment condition was used to determine proliferation rate percentages relative to the untreated cell populations (**Fig. 1A**), the doubling times (**Fig. 1B**), the dividing or mitotic fraction (**Fig. 1C**). Cytotoxicity of rotenone was assayed by determining the percentage of dead cells under each treatment condition, after carefully collecting all detached cells and combining them with the rest of the trypsinized cells prior to counting the cells with using the trypan blue dye exclusion method (**Fig. 1D**).

### GSH, Protein Carbonyls and 8-OH-dG Measurements

Total glutathione (GSH) was measured in rotenone-treated and control cells using a GSH assay kit (Cayman Chemical Co., Ann Arbor, MI) following the manufacturers protocol. GSH was normalized to total cellular protein. For measuring protein carbonyls, cell extracts were lyzed in a mild buffer (10 mM CHAPS, 0.15 M NaCl, 0.01 M NaH_2_PO_4,_ 2 mM EDTA, 200 U/ml DNAse I, 2 µg/ml RNAse and protease inhibitors) and the soluble fraction was collected. The insoluble pellet was homogenized in a stronger buffer (150 mM NaCl, 10 mM NaH_2_PO_4_, 1 mM EDTA, 5% SDS, and 0.5% deoxycholate and protease inhibitors) and the collected soluble fraction was combined with the other fraction and protein was measured by Bradford assay (BioRad Laboratories, Hercules, CA). The oxyblot protein oxidation detection kit (Millipore, Temecula, CA) was used to assess the protein carbonyls following the manufacturer’s protocol using both 20 µg and 10 µg of protein as starting material. The end product was spotted on an immunobilon P membrane (Millipore) and UV light cross-linked. The membrane was incubated with appropriate antibodies and protein carbonyls were detected using the ECL system (Amersham Biosciences, Piscataway, NJ). Dot blots were made in duplicates with samples from 3 different experiments; bands were quantified by densitometry and normalized to non-treated controls. For measuring the 8-OH-dG DNA adduct, rotenone-treated and control cells grown for 4 weeks were challenged with 200 µM H_2_O_2_. DNA from 3 separate experiments was extracted using the DNA Extractor Kit (Wako Chemical, Inc., Richmond, VA). Levels of 8-OH-dG in 50 µg of DNA were measured with an ELISA kit (New 8-OH-dG, JaICA, Fukuroi, Japan) following the manufacturer’s instructions. A standard curve was used to determine the amount of 8-OH-dG in each sample and results were converted to ng of 8-OH-dG per mg of DNA.

### Statistical Analysis

Data was analyzed using the Prism 5.0 software (Graphpad Software Inc., San Diego, CA) by determining the mean and standard errors for each group and performing the one-way Mann-Whitney test (t-test non-parametric; P<0.05) or the Pearson’s correlation analysis test.

## Supporting Information

Figure S1 **Schematic summary of the effects of rotenone exposure on the transcript levels for genes associated with signaling cascades, apoptotic and cytoprotective pathways. Format:** PDF Size: 766 KB; This file can be viewed with: Adobe Acrobat Reader.
(PDF)

Figure S2 **Expression patterns of 58 common differentially-regulated genes (DRGs) by 5 nM rotenone at 4 weeks.** Expression pattern of three transcriptome analysis experiments of SK-N-MC cells chronically treated with 5 nM rotenone or vehicle (0 nM) for 4 weeks (4w). 58 genes of the 898 DRGs detected by dCHIP in Greene et al., [29], data were also differentially-regulated in our 4w5 nM treatment group, as shown in (**A**); for comparison the expression pattern of the same DRGs in Greene et al., [29], data is shown in (**B**), where GSM107862, GSM107863, and GSM107864 correspond to the vehicle-treated samples at 4 weeks and GSM107865, GSM107866, and GSM107867 correspond to the 5 nM rotenone treated samples at 4 weeks. DRGs were clustered by hierarchical average-linkage analysis, as implemented in the MeV software accessible in the TM4 suite [118], and shown in colorgrams depicting the expression level of the genes (rows) in each individual sample (columns). Expression above the mean is displayed in red and below the mean in blue (for normalized scale see bar on top). In (**C**) below, similar clustering analysis of the 58 commonly affected DRGs was applied to the same samples from both studies in order to visualize the distance (blue shade in dendogram) between control and treated samples in both datasets, and the differences in magnitude and direction of changes between datasets under the same scale (top). **Format:** PDF Size: 509 KB; This file can be viewed with: Adobe Acrobat Reader.
(PDF)

Figure S3 **Proliferation kinetics curves.** Cell proliferation curves for populations of rotenone-treated and vehicle-treated cells during exponential growth for a 30 days period of culture. SK-N-MC cells were seeded at a density of ∼3×10^3^/cm^2^ and grown, in the same medium and conditions as for the transcriptome analysis, to a confluence not higher than ∼70% in the absence, and presence of 5 nM and 50 nM rotenone. Data are presented as mean ± S.E.M (N = 3) of the number of cells at each time point; plotted in linear scale (**A**) and in logarithmic scale (**B**). The exponential curve fitting in (A), which assumes that that all cells are actively dividing to give rise to two daughter cells, was performed with the Prism 5.0 software (Graphpad Inc.), using the growth equation : Nt  =  N_0_×2^t/DT^ where N_0_ is the initial number of cells, Nt is the number of cells at time, t, and DT is the division time; and used to determine the growth rate constant, which was subsequently used to ascertain the effects of rotenone on growth rate (**Fig. 1A** **),** the doubling times (**Fig. 1B**), and the fraction of dividing cells (**Fig. 1C**). **Format:** PDF Size: 261 KB; This file can be viewed with: Adobe Acrobat Reader.
(PDF)

Table S1 **Microarray data quality control and differentially-regulated genes (DRGs). Format.** PDF Size: 306 KB; This file can be viewed with: Adobe Acrobat Reader.
(PDF)

Table S2 **Complete lists of differentially regulated genes (DRGs) in cells treated with 5 nM and 50 nM rotenone for 1 week and 4 weeks. Table S2A.** List of 75 DRGs in cells treated with 5 nM rotenone for 1 week; Table S2B: List of 112 DRGs in cells treated with 50 nM rotenone for 1 week; Table S2C: List of 457 DRGs in cells treated with 5 nM rotenone for 4 weeks; Table S2D: List of 619 DRGs in cells treated with 50 nM rotenone for 4 weeks. A gene probe was considered differentially regulated if it reported fold change (FC) >2.0, after pairwise comparison using the model-based expression indexes (MBEI) method implemented in dCHIP [100]. Lists of genes with altered expression across samples were generated by filtering the data using the following criteria in dCHIP: 0.5< sd/mean >1000 (or coefficient of variation, CV) and used for comparison analysis of genes that satisfy the following criteria: FC >2.0 (compared to the corresponding vehicle-treated control), and 90% lower bound of FC, p<0.05 (unpaired t-test) using dCHIP, which produced a preliminary lists of differentially expressed genes comprising 92, 135, 633 and 832 genes in groups 1w5 nM, 1w50 nM, 4w5 nM and 4w50 nM respectively. Redundant probe sets were then removed, retaining the one with the lowest p-value and CV; which reduced the lists of differentially-expressed genes in these groups to 88, 127, 581 and 762 respectively. These gene lists were then corrected for multiple testing using the SAM (Significance Analysis of Microarray) algorithm [117], implemented in the Multi-experiment Viewer (MeV) software accessible in the TM4 suite [118], with a delta value set at >1.2, for a false discovery rate (FDR) or median number of falsely significant genes of 0 (%) 90^th^ percentile. After these corrections, the gene lists were reduced to 75, 112, 457 and 619 in the 1w5 nM, 1w50 nM, 4w5 nM and 4w50 nM groups respectively. Upregulated genes are shaded in pink, whereas downregulated genes are shaded in blue. The probes from these lists were used to identify overrepresented functional categories using DAVID listed in Table 1. **Format:** xls Size: 142KB; This file can be viewed with: Microsoft Excel.
(XLSX)

Table S3 **Rotenone-regulated genes associated to neurological and vascular diseases. Format:** PDF Size: 438 KB; This file can be viewed with: Adobe Acrobat Reader.
(PDF)

Table S4 **Neuroblastoma cells lineage specific markers. Format:** PDF Size: 278 KB; This file can be viewed with: Adobe Acrobat Reader.
(PDF)

Table S5 **Greene et al.,(2008),** [**29**] **array data quality control and differentially-regulated genes (DRGs) analyzed using dCHIP. Format:** PDF Size: 319 KB; This file can be viewed with: Adobe Acrobat Reader.
(PDF)

Table S6 **List of primer sets used for quantitative-real time polymerase chain reaction (qRT-PCR) analysis. Format:** xls Size: 12KB; This file can be viewed with: Microsoft Excel.
(XLSX)
