# Supplementary material for: Transcriptome Analysis of a Rotenone Model of Parkinsonism Reveals Complex I-Tied and -Untied Toxicity Mechanisms Common to Neurodegenerative Diseases
Source: PLoS One. 2012 Sep 7;7(9):e44700. doi: 10.1371/journal.pone.0044700 (PMC3436760; doi:10.1371/journal.pone.0044700)
Supplement: Figure S1 — Schematic summary of the effects of rotenone exposure on the transcript levels for genes associated with signaling cascades, apoptotic and cytoprotective pathways. Format: PDF Size: 766 KB; This file can be viewed with: Adobe Acrobat Reader. (PDF) [file pone.0044700.s001.pdf]

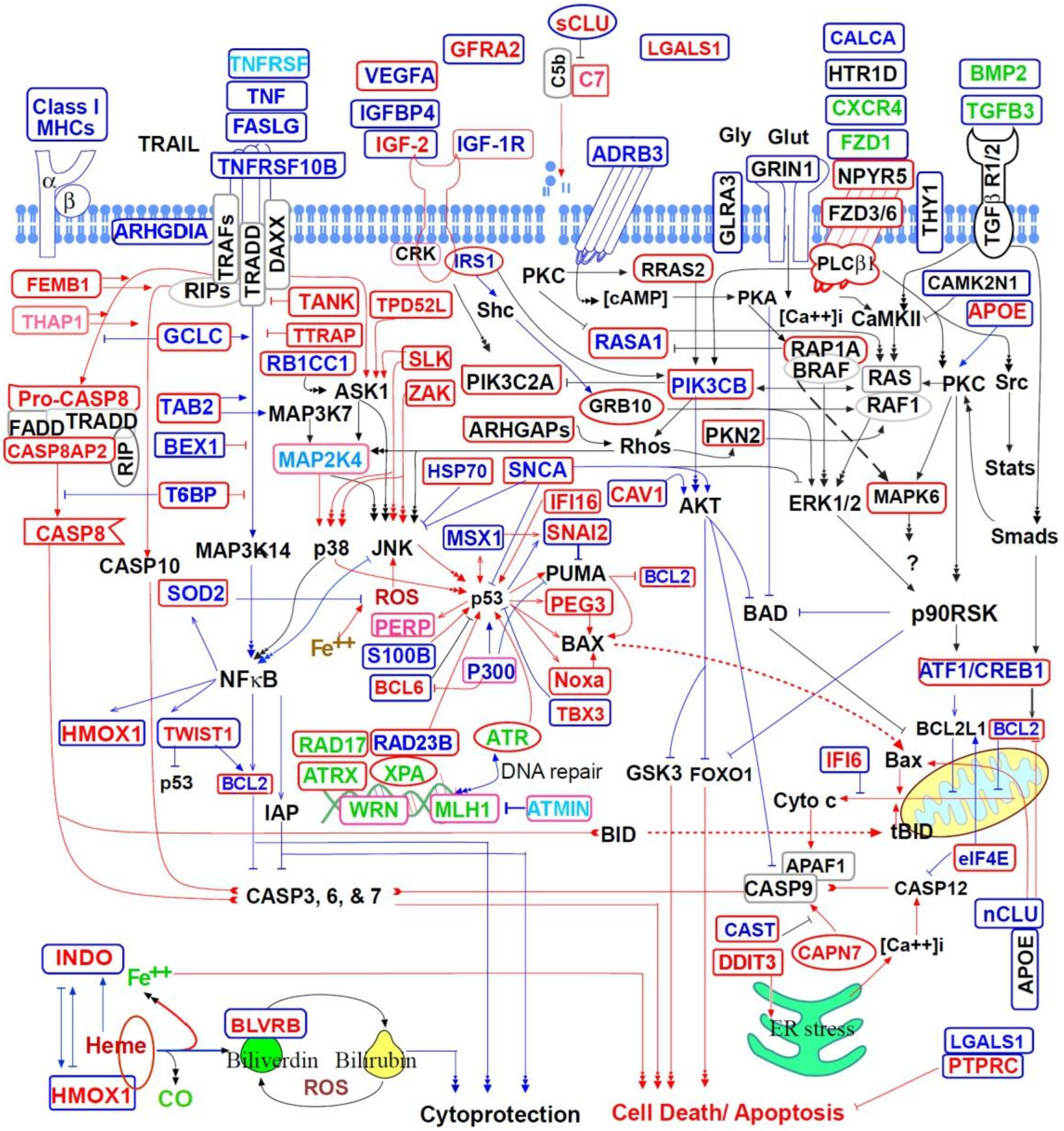

Figure S1. Schematic **summary of the effects of rotenone exposure on the transcript levels for genes associated with signaling cascades, apoptotic and cytoprotective pathways**.

Notes: Genes up- and down-regulated by rotenone are indicated in red and blue bordered boxes respectively; genes whose change is potentially detrimental to cells are shown in red letters (or pink for those affected only by the 5 nM dose) and those whose change is potentially beneficial to cells are shown in blue letters (or light blue for those affected only by the 5 nM dose); certain genes (e.g. *HSPA1A*, *MLH1* and *PTPRC*) were differentially affected by both doses and thus have opposite effects depending on the dose; ambivalent genes that may exert positive and negative regulatory effects are shown in green letters and certain genes (e.g. nuclear (nCLU) and secreted (sCLU) clusterin) may have ambiguous effects depending on their localization. Blue arrows and blunted-lines represent potentially protective effects, while red arrows represent potentially detrimental effects. Genes in black letter, in non-filled boxes represents apoptosis intermediators genes. Genes in black letters (without boxes) were not significantly altered by rotenone but are affected by genes that were or are included solely to facilitate the pathway visualization. The complete list of apoptosis regulatory genes is given in Figures 3 and 4. The expression of the anti-apoptotic *PTPRC* and *HPA1A* and the pro-apoptotic *MHL1* genes was ambiguously affected at 4 weeks by both doses; thus exhibiting opposing expression pattern among the 2 rotenone doses. The following discussion is intended to provide an explanation as to how some of these genes could be involved in mediating the cytotoxic effects of rotenone and the cytoprotective to counteract such rotenone effects by highlighting their known associations with cell survival and apoptosis and at the same time provide the references to the works suggesting their involvement in these cellular processes.

Evaluation of the functions genes whose expression was changed by rotenone after 4 weeks reveals the extrinsic apoptosis pathways as a mediator of the response to chronic rotenone exposure. This is indicated by the significant increase of the *CASP8* and *CASP8AP2* transcripts and other genes that participate in this pathway and other pathways that effect cell death mediated through this pathway.

The apoptosis extrinsic pathway (review in [1] is mainly mediated through death receptors (DRs), a subfamily of the tumor necrosis factor (TNF) receptor superfamily (TNFRS), including TNFRSF1A/B, Fas, TNFRSF10AB, and NGFR (Nerve growth factor receptor). The decision between cell death and survival in the DR-mediated signaling pathways is dictated by the distinct complex formed between various death domain (DD-) and death effector domain (DED)-containing adaptor molecules [2]. For instance, TNFR1 does not induce cell death spontaneously; instead, it activates NF-κB, which triggers a pro-survival signaling pathway [1]. In the context of the TNFR1 its activation leads to the rapid assembly of complex I, at the plasma membrane, containing TRADD (TNFR1-associated death domain protein), which recruits other adaptor proteins such as TRAF2 (TNF receptor-associated factor 2), and RIP1 (receptor-interacting protein 1) kinase [3]. RIP1 can then interact with IKK (IκB kinases) complex enabling the ubiquitin-targeted degradation of IκB by the proteasome, the release of activated NF-κB and its translocation to the nucleus where it activate survival genes including *CFLAR* (CASP8 and FADD-like apoptosis regulator), *IAPs* (Inhibitor of apoptosis proteins), *BCL2*, *BCL2L*, and *SOD2*. Subsequently, the TRADD-RIP1-based complex dissociates from the plasma membrane and recruit FADD (Fas-associated death domain proteins), which in turn binds procaspase-8 and procaspase-10, to form complex II, which promotes auto-activation of both apoptosis initiator caspases. CFLAR, an inhibitor of CASP8, maintains the balance of the effects by complex I versus complex II. Thus, the apoptotic effect of TNF is only manifested when activation of NF-κB, by complex I, is inadequate. Activated CASP8 can promote apoptosis via two parallel cascades depending on the cell type: it directly cleaves and activates CASP3 with the subsequent activation of other effector caspases (CASP6 and 7), and it indirectly activates CASP3 via cleavage of BID (BH3 interacting domain death agonist) to generate activated tBID (truncated BID). tBID in turns activates the mitochondrial apoptotic mechanism by inducing cytochrome c release, which activates APAF1 that in turn leads to activation of other effector caspases, thus merging the extrinsic pathway to the intrinsic pathway both ultimately leading to apoptosis (see reviews [1, 2]), or necrosis, which diverge from apoptosis immediately downstream of TRADD, with FADD/CASP8 promoting apoptosis and RIP1 promoting necrosis [4]. RIP1 also activate the NF-κB-activating kinase (NIK) in the non-canonical NF-κB pathway, which activates the IKK complex (reviewed in {Danial, 2004 #2129;Jin, 2005 #2119;Falschlehner, 2007 #2077). The intrinsic pathways are initiated inside cells by a variety of stimuli, including ROS, DNA damage, endoplasmic reticulum (ER) stress, and many other insults. Key events of these pathways, which lead to mitochondria outer membrane permeabilization (MOMP), are mainly mediated by the Bcl-2 family of proteins, including the pro-apoptotic BCL2-agonist of cell death (BAD), and BCL2-associated X protein (BAX), and the anti-apoptotic BCL2 and BCL2L1 (aka BCL-XL), which lead to the positive and negative regulation of the release of cytochrome c, and several other apoptosis effectors. The functions of the pro-apoptotic molecules is counteracted by the pro-apoptotic members and the pro-apoptotic family members are activated by members of the third subfamily, the BH3-only proteins, which include BID, Puma and Noxa (official name: PMAIP1). Cell death pathways and apoptosis pathways in particular are closely interwoven with other essential cellular processes pathways. Thus, several DR-ligands signaling does not only lead to the activation of NF-κB or the caspases cascade, but can also enable or disable other pathways, including the activation or inhibition of mitogen-activated protein kinases (MAPKs) signaling pathways that influence cell fate by regulating cell development, proliferation, survival and death. Furthermore, besides DRs, other factors in neuronal cells can activate the NF-κB pathway including, other cytokines, neurotransmitters (e.g. glutamate), and oxidative stress (ROS) by coupling this pathway to kinase cascades such as Ca++/calmodulin-dependent kinase II (CAMKII), AKT, PKC, and MAPKs (reviewed in [5]).

The MAPKs family of proteins comprise three subfamilies: the extracellular signal-regulated kinases (ERKs), the c-Jun N-terminal kinases (JNKs; aka SAPKs (stress-activated protein kinases)), and the p38-MAPK (p38) [6-8]. MAPKs enable a variety of cellular processes including inflammation, cell proliferation and differentiation as well as apoptosis. Although activation of each MAPK subfamily family have their own features and vary on the initial triggering factor, they are all activated by the serial phosphorylation of hierarchically assembled, MAP kinase modules, comprised of MAP3K-MAP2K-MAPK, in order to generate a proper biological response [1, 6, 8]. JNK and p38 pathways are more responsive to stress stimuli, including survival factor withdrawal, ROS and other environmental stresses, and inflammatory cytokines, growth factors and GPCR agonists. Both pathways utilize MAP3Ks such as the Apoptosis Signal-regulating Kinase 1 (ASK1; a.k.a. MAP3K5), MAP3K1 and ZAK (a.k.a. MLK7), and MAP2Ks such as MAP2K4 and/or MAP2K7, which activate the JNKs or p38s. ASK1-induced apoptotic activity is enhanced through direct protein-protein interactions by two death receptor-associated proteins, DAXX and TRAF2 in the Fas and TNF-α pathways, respectively. While the activation of p38 requires RIP and TRAF2, the stimulation of JNK is independent of them. Activation of JNK or p38 signaling cascades, leads to gene expression regulation through transcription activation factors and co-repressors including AP-1 (includes c-jun), p53, ATF2, and STAT3, that ultimately leads to production of apoptosis regulatory proteins such as transcription factors themselves (p53), Bcl-2 family proteins, and cytokines (TNF, FasL). DNA damage response involves the triggering of the p53 apoptosis pathway, which induces the expression of several pro-apoptotic factors including BAD, BAX, Puma, and PMAIP1 (Noxa), whereas STAT3 induces the expression of pro-survival proteins BCL-2 and BCL2L1. Stress signals are also delivered to the JNK signaling cascade by small GTPases of the Rho family (Rac, Rho, cdc42). The JNK pathway may play a pro-apoptotic role in neurons by disabling anti-apoptotic BCL2 and BCL2L1 and by enabling pro-apoptotic the BH3-only protein member BIM (see review [1]). The ERK cascade is usually activated in response to growth stimuli and GPCR ligands [6]. Their activation through DRs is mainly associated with an anti-apoptotic function [9]. This cascade comprises the MAP3Ks (including RAF1 and BRAF), the MAP2Ks (MEK1/2) and the MAPKs (ERK1/2). Activation initiated by receptors such as RTKs, GPCRs and ion channels transduce stimulatory signals through the RAF/MEK/ERK module via distinct RAS isoforms. Particular components of the cascade depend on the initial stimuli. They usually involve a set of adaptors (e.g. IRS1, GRB10, CRK) and a small GTP binding protein (e.g. RAS, RAP1A), which activates the core module of the cascade. Activated ERK complex regulate targets in the cytosol or translocate to the nucleus where it regulates the transcription of genes involved in biological responses such as growth, differentiation, and development (review {Roux, 2004 #2102). ERK activation suppresses TRAIL-mediated apoptosis and inhibits the processing of CASP8 and BID [9]. Induction of anti-apoptotic Bcl-2 proteins is achieved through the stimulation of the ERK1/2 and PKC pathways that leads to activation of p90RSk, which in turn activates CREB and induces the expression of BCL2 and BCL2L1. The PKB/Akt (hereafter AKT) cascade is triggered by stimuli that activate diverse cell surface receptors including, RTKs, cytokine receptors, and GPCRs, leading to production of phosphatidylinositol 3,4,5 triphosphates (PtdIns(3,4,5)P3) by phosphoinositide 3-kinase (PI3K). Activation of the PI3K-dependent cell-survival response leads to activation of AKT isoforms, which mediate several of the downstream events regulated by PI3K such as inhibition of pro-apoptotic BAD (Bcl-2 antagonist of cell death), CASP9, GSK-3 (glycogen synthase kinase-3), IKKβ, and FOXO family members (reviewed in [10, 11]). NGF can both induce and inhibit apoptosis, depending on the cell type examined. NGFR transduce its signal by interaction with TRADD, TRAF2, TRAF6, and the RIP2 kinase,

which differentially regulate both the activation of the transcription factor NF-κB and the anti-apoptotic effect of NGF [12]. Ligands such as insulin, and IGF1/2 activate IGFR1, which tyrosine-phosphorylates and activates docking molecules such as IRS1 that interact with SH2 domain-containing proteins including phosphatidyl-inositol 3-kinase (PI3K) and growth factor receptor-bound protein 10 (GRB10), that link this receptor signaling to MAPK pathways. Activated IGFR1 triggers various MAPK-mediated signaling pathways. Cellular response to NGF is also elicited via the specific tyrosine kinase receptor, type 1 (NTRK1), an RTK type receptor similar to the IGFR1 [13]. GPCRs are activated by a wide variety of external stimuli. Ligand binding activates the receptor, triggering diverse signaling cascades. Receptors coupled to different G-protein subtypes (Gs, Gq, Gi, or G12) activate the small G-protein/MAPK cascade, utilizing different tyrosine kinases. GPCRs can also use PLCβ to mediate stimulation of PKC and CaMKII, which leads either, to activation or inhibition of the downstream MAPK pathway (reviewed in [14]. Src kinases are recruited by activation of PI3Kγ, by receptor internalization or cross-activation of receptor tyrosine kinases. In summary, parallel and distinct signaling pathways, triggered by death stimuli, induce the activation of NF-κB, AKT and MAPKs, which allows for a functional dichotomy leading to cell survival and apoptosis [1, 6, 8].

Among those genes whose up-regulation by rotenone may be detrimental to the cells, the following are noteworthy: *CASP8* and *CASP8AP2* both were induced by rotenone and encode for proteins components of the DISC. CASP8AP2 is required for the activation of CASP8 in DR-mediated apoptosis and is involved in TNFα-induced activation of NF-κB [15, 16]. CASP8 may play a role in neurodegeneration [17]. Two pro-apoptotic TRAFs regulators, *TTRAP* and *TANK* were induced by rotenone. TTRAP in association with TRAFs, but not with TRADD, regulates signal transduction by several TNFRSF members and inhibits NF-κB activation [18]. TANK binds to and sequesters various TRAFs, thus disabling them, and precluding TRAF-mediated NF-κB activation signaled by receptors CD40, TNFRSF1A and TNFRSF1B [19]. *FEM1B*, was also increased by rotenone (50 nM), and its over-expression has been shown to induce apoptosis in mammalian cells [20]. *ZAK*, also up-regulated by rotenone, is a stress-activated component of the MAPK signaling cascades JNK and p38, which as a MAP3K mediates pro-apoptotic signaling through the JNK and p38 pathways [21, 22]. *TPD52L1*, induced by the 50 nM dose, promotes apoptosis through ASK1 [23]. *THAP1*, increased by rotenone (5 nM), is a nuclear pro-apoptotic factor associated with pro-myelocytic leukemia nuclear bodies, which enhances TNFα-triggered apoptosis and been linked to neurodegenerative diseases [24]. *BTG1*, increased by rotenone (50 nM), has been shown to be a BCL2-regulated mediator of apoptosis in breast cancer cells [25]. *ATG5*, induced by rotenone (50 nM), has been shown, both *in vitro* and *in vivo*, to promote mitochodria-mediated apoptosis [26].

Additional rotenone-induced pro-apoptotic genes include: Increased expression of the GC kinase gene *SLK*, which mediates TNFα- cMyc- and UV-induced apoptosis via activation of the JNK signaling pathway [27]. and is an anoxia/recovery-dependent kinase that signals through ASK1 and p38 to induce apoptosis via activation of CASP8 and CASP9 and also attenuates the protective features of the ER stress response, which may add to its pro-apoptotic [28]. CAPN7, induced by rotenone (50 nM), is a crucial mediator of apoptosis. Mitochondrial dysfunction leads to disruption of Ca++ homeostasis in the cytosol enabling the activation of Ca++ -dependent enzymes such as calpains. Recent studies support the involvement of increased calpain and CASP3 activities in rotenone-induced neurotoxicity in spinal cord of Lewis rats [29] and calpain involvement in rotenone-induced apoptosis has recently been shown in cultured neurons [30]. The combined up-regulation of CAPN7, CASP8 and CASP8AP2 is interesting as they are among the main enzymes that control activation of CASP3 and extrinsic pathway [1, 15, 29]. *FDX1,* increased by rotenone (5 nM), is a component of a mitochondrial cytochrome P450 system, which consists of two more protein entities besides FDX1: ferredoxin reductase and CYP11B2 (down-regulated by rotenone), which is a source of mitochondrial ROS production, and play a role in the induction of mitochondrial apoptosis [31].

The response to rotenone exposure appears to interfere with and/or involve p53-dependent apoptotic pathways as indicated by the up-regulation of at least three p53-target genes that mediate apoptosis and various other genes that intervene directly or indirectly in the p53-mediated apoptosis. These include: PEG3, which regulates the TNF response by associating with TRAF to activate NF-κB [32]. In addition, following DNA damage, PEG3 is induced by p53 and serves as an intermediary between p53 and BAX to promote neuronal cell death [33]. *PMAIP1* (*Noxa*) is another p53-induced gene also induced by rotenone, belongs to the pro-apoptotic BH3-only subfamily of the Bcl-2 protein family (which includes PUMA, BID, BAD, and BNIP3), that are activated by a variety of intracellular and extracellular stress signals [34-36] and act as initiator of apoptosis by relaying such signals to other pro-apoptotic and anti-apoptotic Bcl-2 family members, such as BAX, BCL2, and BCL2L1. Multiple studies identify *Noxa* and *PUMA* as regulators of p53-dependent and -independent apoptotic cell death after genotoxic injury, playing a role that is markedly cell-type and stimulus specific [37, 38]. Noxa is the prevalent mediator of apoptosis induced in motor neurons after axotomy, [34, 39]. Reported evidence indicates that besides PUMA, p53 and Noxa are also components of the ER stress-induced apoptotic pathway in MEFs [40]. Noxa has been shown to participate in the Ca2+/calpain-independent pathways of MOMP induction, and required E1A expression for cells to become susceptible to MOMP induction [36]. In contrast to Noxa, over-expression of the third rotenone-induced p53-target gene *PERP* alone is reported to be sufficient to cause apoptosis in multiple paradigms, including the developing CNS, suggesting that PERP represents a critical transducer of the signal that favors apoptotic cell death [41]. Besides these recognized p53-targets, other genes affected by rotenone are linked to the p53 pathways. For instance, *ATR,* which is activated by UV light and chemical genotoxins-induced DNA double strand breaks (DSBs) damaged, was up-regulated by rotenone. ATR is a protein whose downstream signaling targets determine if cells will undergo cell-cycle arrest, DNA repair or apoptosis and since repair of DSBs is a highly important anti-apoptotic mechanism, ATR is regarded primarily as an apoptosis inhibitor [42]. However, at high DSBs levels ATR can activate sufficient p53 protein and induce pro-apoptotic genes. Thus, it should be noted that the decision between cells survival or death depends on the level of ATM/p53 (and ATM) activation and not on the transcript levels of any of these p53 machinery components [42]. *IFI16,* also rotenone-induced, is a BRCA1-associated protein involved in p53-mediated apoptosis and inflammation signaling cascades [43]**.** On the other hand, the *BCL6* proto-oncogene, decreased by rotenone, is a nuclear transcriptional repressor, with roles in survival, which is inhibited by EP300 (E1A binding protein p300, also induced by rotenone; aka p300) [44]. *EP300*, an anti-apoptotic gene, has been shown to be a key regulator of the p53 response [45]. Two genes with antagonistic regulatory effects on p53 function, *SNAI2* and *MSX1*, were down-regulated by rotenone. The transcriptional repressor SNAI2 was found to be nearly as active as BCL2 or BCL2L1 in promoting the survival of IL-3-dependent murine pro-B cells deprived of the cytokine [46]. Interestingly, *snai2* and *msx1* have been identified as early regulatory factors in the induction of neural crest in *Xenopus,* where these two genes exert opposing effects on apoptosis with snai2 acting as an anti-apoptotic protein, and msx1 promoting cell death; thus, controlling the pattern of programmed cell death [47].

MSX1 has also been found to play a similar pro-apoptotic role in adult tissue, where it has been shown to interact, stabilize, and regulate p53 function by controlling its localization [48]. *TP53I11*, which was decreased by rotenone (50 nM), is another p53 target gene, and its over-expression has been shown to induce ROS production and apoptosis [49]. *APC*, also up-regulated by rotenone (50 nM), participates in Wnt signaling and regulates many cellular processes including cell cycle progression, cell migration, cell adhesion, and cell fate determination. Expression of APC in colorectal cancer cells containing inactive *APC* alleles leads to significant reduction of cell growth resulting from the induction of apoptosis [50].

Among those genes whose down-regulation by rotenone may be detrimental to the cells, the following are noteworthy: *TBX3,* decreased by rotenone (50 nM), is an anti-apoptotic p53-interfering gene, which is as an important mediator of WNT/β-catenin activities on cell proliferation and survival [51]. *CTSB*, another p53-regulator gene that was down-regulated by rotenone (50 nM), is a cysteine protease involved in intracellular proteolysis, which has been identified as a contributor to the apoptotic phenotype of cystatin B-deficient mice [52]. However, *CtsB* (-/-)/*CtsL* (-/-) mice present severe neurodegeneration due to select massive neuronal apoptosis, suggesting a crucial role for these cathepsins in CNS maintenance [53]. *GFRA2*, down-regulated by rotenone (50 nM), encodes for a receptor component of the GDNF-family receptors complex, formed by the TKR Ret and one of the GFRA-glycosyl-phosphatidyl-inositol (GPI)-linked receptors, which determines ligand specificity. GFRA2 ligands include **g**lial cell line-derived neurotrophic factor (GDNF), and neurturin, which besides serving as a trophic factor for midbrain dopaminergic neurons, have been implicated in diverse processes including nigral dopaminergic neuronal survival. However, the effectiveness of these receptors and factors largely depends on their availability after damage. For instance, evaluation of the role of GDNF in motoneurons (MNs) survival in mice revealed that many GDNF-independent MNs are characterized by the presence of GFRA2, which indicates that the GDNF-independent population represents MNs that require other GFRA2 ligands, such as neurturin, for their survival [54]. Reported evaluation of the expression pattern of these receptors in SNpc neurons, in a rat model of PD using 6-hydroxydopamine (6-OHDA) as the insult, indicated that GFRA2 expression was differentially regulated as a transient and compensatory response to 6-OHDA damage decreased only after 6 days of the insult [55]. Thus, the decreased expression of GFRA2 induced by chronic exposure to rotenone deprives these cells of a major survival pathway. *APOE*, down-regulated by both rotenone doses, is a multifunctional molecule that exists in humans as one of three common isoforms, E2, E3 or E4, and plays a fundamental role in the relocation of lipids among cells. Indeed, APOE4 may be detrimental in the process and is a constituent of amyloid plaques. Besides AD, APOE4 is associated with various neuropathological processes, including stroke, PD, amyotrophic lateral sclerosis, multiple sclerosis, and CNS ischemia. Multiple studies have shown that, compared to APOE3, APOE4 is selectively toxic to neurons *in vitro,* as well as, *in vivo* [56, 57]. The neurotoxic effect of APOE4 have been shown to be mediated via dysregulation of Ca++ homeostasis [58]. Increased APOE expression has been associated with apoptosis in non-neuronal cell types [59], and recent studies in neuronal cell lines also report similar increased levels of APOE that correlate with apoptosis and suggest that APOE may play a role in the clearance of apoptotic bodies [60]. The gene for the extracellular matrix glycoprotein, fibronectin 1 (*FN1*), was down-regulated by rotenone. FN1 regulates a variety of cellular processes including cell survival and growth; alterations in the matrix or in matrix survival signals can trigger apoptosis [61]. Also reduced by rotenone were *CAV1*, which has been shown to act as a cell survival enabler by up-regulating the activation of the pro-survival AKT pathways [62]; VEGFA, which has been shown to inhibit endothelial cells apoptosis both in vivo and in vitro [63, 64]; *IFI6*, an interferon inducible gene, whose protein localizes at mitochondria, and exhibits anti-apoptotic function through inactivation of CASP3 [65]; *TIMP*, a multifunctional matrix metalloproteinase (MMP), tissue inhibitor that, in conjunction with MMPs, regulates the remodeling of extracellular matrix and inflammation. Downregulation of *TIMP1* in the CNS has been recently associated with several neurological diseases involving chronic neuroinflammation [66]. In addition, TIMP1 has been shown to inhibit the intrinsic, as well as, the extrinsic apoptosis pathway, triggered by a variety of insults in breast epithelial cells [67].

A final group of genes whose rotenone-induced changes could have detrimental consequences to the cells comprises genes directly involved in heme degradation, iron homeostasis, and cellular redox state. These included the reduced expression of *HMOX1*, whose encoded protein, HO-1, is an important factor in the response to oxidative stress, generally regarded as a very sensitive marker of cellular oxidative stress [68]. Downregulation of *HMOX1* may have contrasting effects on the redox balance of SK-N-MC cells because during heme degradation it generates bilirubin, carbon monoxide (CO) and Fe2+, which all affect redox homeostasis in contrasting manner [69]. Bilirubin is a potent cytoprotectant, but when high it induces apoptosis [69]; whereas Fe2+ and CO exacerbate intracellular OS [70]. Hence, under chronic rotenone toxicity, *HMOX1* repression may, in the short run, be a compensatory as it lessens the effects of Fe2+ and CO on OS. A similar adaptive response occurs in endothelial cells exposed to CO [71]. However, low HMOX1 levels may impede the cells ability to mount a surge response to other insults and deny them of pro-survival options under further stress conditions, such as previously seen with H_2O_2 in this model [72]. Several additional genes, with possible roles in protecting cell from oxidative damage were affected by rotenone. Among them, *FTH1* was decreased by both rotenone doses and the *BLVRB* (Biliverdin reductase B) gene was reduced by rotenone (50 nM). By its ability to sequester free iron, FTH1 is regarded as the primary iron storage protein in cells, and is a potent antioxidant that combats ROS accumulation [73, 74]. In many instances, impending toxicity derived from the intracellular release of heme-derived ferrous iron is circumvented by co-induction of FTH1synthesis, a [68, 75]. Thus, if co-induction of FTH confers protection by providing a sink for free ferrous iron, reduced expression of *FTH1* may impair iron homeostasis and contribute to the neuroendangering aspects of rotenone. BLVRB converts biliverdin to bilirubin in the fetus, and catalyzes the reduction of several non- isomers of biliverdin, flavins, methemoglobin, and ferric ion in the adult [76]. Therefore, the down-regulation of these two genes in conjunction with the down-regulation of *HMOX1,* suggest that besides impairment of heme metabolism in these cells, the homeostasis of heme derived elemental iron and consequently, ROS may be primarily affected in the response to rotenone. The decreased expression of *INDO* after exposure to rotenone lends further support to this notion. INDO is the rate-limiting enzyme in the catabolism of tryptophan. As such INDO controls, not only the availability of this amino acid, but also the generation of oxidative metabolites that mediate selective apoptosis, which imparts INDO a critical immmunoregulatory role in eliciting a protective immune response through the modulation of the inflammatory process [77]. Interestingly, HO-1 and INDO have been shown to co-regulate each other through the modulation of heme availability by HO-1 and oxidative stress mechanisms [78]. Noteworthy however, besides causing apoptosis, ROS are emerging as key effectors in signal transduction [79]. In fact, recent evidence established that the NF-κB achieves its anti-apoptotic activity in part by negatively modulating the JNK pathway and by suppressing the accumulation of ROS. The reciprocal negative control that NF-κB applies on ROS and JNK signaling was found to be required for antagonism of apoptosis elicited by TNF-α and likely other stimuli [4]. Both *FTH1* and *TWIST1*, also down-regulated by rotenone, have been identified as key effectors

of this antagonistic cross-talk between these pathways [80, 81]. TWIST1 mediates the protective function of NF-κB, and halt apoptosis induced by diverse stimuli without interfering with cytotoxic JNK, and p53 signaling [81].

At least three genes encoding for proteins with major recognized antioxidant neuroprotective functions were up-regulated by rotenone. The first gene, *SOD2,* is a mitochondrial antioxidant enzyme that modulates the cellular redox environment by serving as the first line of defense against ROS. Induction of this gene by rotenone has been recently corroborated at the mRNA and protein levels [82]. The second major up-regulated antioxidant gene is that of the glutamate-cysteine ligase catalytic (GCLC) subunit, a component of the heterodimer enzyme GCL, which is the rate-limiting enzyme in glutathione (GSH) synthesis [83, 84], and thus regulates GSH homeostasis. Enhanced expression of GCLC has been shown to inhibit the activation of the NF-κB, JNK, and ERK signaling cascades mediated by various stimuli including TNF and to abrogate TNF-mediated CASP3 activation and ensuing apoptosis [83]. The third major pro-survival gene induced by rotenone was the protein α-synuclein (*SNCA*), which is the molecular hallmark of several neurodegenerative disorders including Parkinson disease (PD). The upregulation of SNCA by rotenone has been previously reported [72] and its competence as an antioxidant molecule capable of preventing the oxidation of unsaturated lipid has recently been reported [85]. SNCA may play a regulatory role at the synaptic terminal, by being involved in either handling pre-synaptic vesicles [86] and/or presynaptic dopamine recruitment [87]. Multiple studies suggest a role for physiological concentrations SNCA in neuroprotection against stress signals-induced apoptosis. Neuroprotection in a variety of neuronal cells, including SK-N-MC, is presumably achieved by reducing CASP3 and p53 expression, and mediated via activation of the PI3K/AKT signaling pathway, which may be lost after an over-expression [88-92]. Neuroprotection via inactivation of the pro-apoptotic JNK signaling pathway has also been detected [93]. In addition, SNCA has been shown to bind ERK and inhibit its activity and signaling cascade [94]. More compelling evidence of a role for SNCA in neuroprotection was obtained with *in vivo* rodent models of apoptotic death in striatum and substantia nigra (*SN*) neurons [95]. Thus, in the normal brain, SNCA may have a neuroprotective function by increasing the availability of pro-survival molecules and decreasing the availability of pro-apoptotic molecules. In normal *SN* dopaminergic neurons, besides this neuroprotective effect, the non-aggregated, physiological form of SNCA is able to diminish the amount of dopamine in nerve terminals, thereby limiting its conversion to ROS and the resulting oxidative stress. Nevertheless, since diverse types of neurotoxins including MPTP and rotenone increase SNCA expression in the brain [96-98], very likely as a results of increased oxidative stress [97], it has been suggested that such an increase in SNCA represents an adaptive homeostatic regulatory response to neurotoxic stimuli [99]. SNCA expression has been associated mainly with cell survival strategies [89].

Besides these three major pro-survival genes, other anti-apoptotic genes were induced by rotenone. Noteworthy, at least three of these genes have been shown to be involved in liver organogenesis. Thus, targeted disruption of *MAP3K7IP2* (aka TAB2) led to embryonic death due to liver degeneration and apoptosis in the mouse, suggesting that MAP3K7IP2 is an essential protein to prevent liver apoptosis during embryonic development [100]. The second gene *MAP2K4*, which encodes a stress signaling kinase that directly activates JNKs in response to a variety of cellular stresses, was targeted-disrupted in the mouse and shown to be required to deliver a crucial and specific pro-survival signal during hepatogenesis and liver formation [101]. The third, *RB1CC1*, which has been shown to negatively regulate apoptosis [102]. Another pro-survival gene, *eIF4E*, was induced by both rotenone doses. This gene has been shown to promote cell survival by contributing to regulation of integrated crucial ER-mediated checkpoints for apoptosis at the cap dependent translation apparatus [103]. In addition, AKT activation through IGFR1 (induced by rotenone) increases the survival of striatal neurons lost during the degeneration in Huntington's disease [104]. The high induction (~3 fold) of the protective gene the *HSPA1A* by the 5 nM rotenone dose is probably partly responsible for the higher survival rate observed for this treatment group. In contrast, *HSPA1A* was among the most markedly repressed genes by the 50 nM dose. Induction of HSPA1A, to some extent, by acute rotenone exposure has been reported at both mRNA and protein levels [82]. HSPA1A is an anti-apoptotic protein that regulates different signaling pathways depending on the cell type and applied stimuli. Thus, HSPA1A has been shown to inhibit JNK-mediated apoptosis [105], and to block the TNFα-induced apoptosis by interfering with the extrinsic pathway. It also inhibits FasL-induced apoptosis by blocking the intrinsic pathway [106]. The expression of *CAST*, the gene coding for the physiological inhibitor of calpain, calpastatin, was also increased in rotenone treated cells. Stimuli that cause an increase in Ca++ influx activate proteases such as calpains and caspases, which are essential for the execution of programmed cell death in diverse cells and models such as spinal cord (SC) injury. Significant higher expression levels of CAST, in juvenile rats than adult rats after Spinal cord (SC) injury has been reported, which correlated with elevated resistance to calpain-mediated proteolysis and neuronal apoptosis in juvenile rats. Besides indicating, that higher CAST levels circumvent calpain activity and thus apoptosis; these results also suggest that age may play a role in SC injury tolerance [107]. However, It has also been reported that caspases may cleave calpastatin and thus, regulate calpain activity during apoptotic cell death [108]; and more recently, that calpain-mediated calpastatin degradation plays a crucial role in regulation of caspase-3 activation during apoptosis [109]. *CREB1* and *ATF1* expression was induced by rotenone, both are members of the CREB/ATF family, which are involved in in cAMP- and calcium-induced transcriptional activation. Both of these proteins have been shown to act in concert as survival factors *in vivo* during early embryonic development in mice [110]; as well as *in vitro* with human cancer cells [111].

Several genes encoding for proteins that promote cell death were down-regulated by rotenone including: LTA (aka TNFβ), a cytokine member of the TNF superfamily that mediates a variety of inflammatory, immuno-stimulatory, and antiviral responses, and plays a positive regulatory role in apoptosis by binding to TNFRSF1A, TNFRSF1B and TNFRSF14 and TNFRSF3 [112]. *TNFRSF10B*, was down-regulated by the 50 nM rotenone dose only, and its protein mediates apoptosis initiated by TRAIL binding through activation of CASP8 [113]. *S100B*, codes for the glial-derived, calcium-binding, protein S100B that exerts paracrine and autocrine effects on neurons and glia, and has functions in cellular energy metabolism, cytoskeleton modification, cell proliferation, neuron and glia differentiation, as well as interactions with multiple immunological processes in the brain. The S100B protein binds to different p53 domains and disrupts p53 tetramerization, thus regulating its subcellular localization [114]. S100B is neuroprotective if it is maintained inside the cells at physiological levels. Otherwise, once it is secreted, its protective or detrimental effects are governed by the local concentration. At the nanomolar range it is pro-neuronal survival during development, and protect primary rat hippocampal neurons against NMDA toxicity by activation of NF-kB [115]. At the micromolar range, it induces pro-inflammatory cytokines and apoptosis. *In vivo* animal studies indicate that elevated levels of S100B in the brain can be detrimental and may promote pathogenic responses to injury by stimulating neuroinflammatory processes, and cause behavioral instabilities and cognitive deficits [116]. BEX1 is a adaptor-like protein that interacts with the NGFR, and was down-regulated by rotenone (5 nM). BEX1 serves as a link between neurotrophin

signaling, the cell cycle, and neuronal differentiation. Its down- regulation in neurons allows cell proliferation under growth arrest conditions and diminishes neuronal differentiation in response to nerve growth factor (NGF); while its over-expression inhibits the induction of NF-κB activity by NGF without affecting activation of ERK1/2 and AKT. RIP2 was found to compete with BEX1 for binding to the NFGR, and to inhibit BEX1 over-expression effects [117]. *PTPRC* was down-regulated by the 50 nM rotenone dose (and increased by the lower dose). It encodes a tyrosine phosphatase essential for efficient antigen-receptor signaling, which mediates apoptosis inhibition, and modulates an array of immune responses, including antigen-receptor signal transduction, T-cell-mediated cytotoxicity, regulation of response to cytokines and cytokine production, and inflammation [118]. In conjunction with the down-regulation of a significant number of major histocompatibility complex (MHC) class I genes (HLA-B, C, F, and G), it suggest that the T-cell cytotoxicity and the neuroinflammatory response cascades may be compromised by chronic exposure to rotenone. This may preclude a response to the elevated levels of the immune response apoptosis-inducer gene *IFI16* [43], in cells exposed to rotenone (50 nM), whereas the increased expression of *PTPRC* in cells exposed to 5 nM rotenone, may confer these cells enhanced survivability. *CALCA* (Calcitonin/Calcitonin-related polypeptide α), whose expression decreased by rotenone (50 nM), is a neuropeptide produced by cells in the CNS, PNS (peripheral NS), and in endocrine cells. CALCA exerts its biological effects on several systems including the cardiovascular, gastrointestinal, respiratory, immune and nervous systems. CALCA has been shown to enhance apoptosis by inhibiting the NF-κB activation [119, 120].

References

1. Jin Z, El-Deiry WS (2005) Overview of cell death signaling pathways. Cancer Biol Ther 4: 139-163.
2. Danial NN, Korsmeyer SJ (2004) Cell death: critical control points. Cell 116: 205-219.
3. Micheau O, Tschopp J (2003) Induction of TNF receptor I-mediated apoptosis via two sequential signaling complexes. Cell 114: 181-190.
4. Papa S, Bubici C, Zazzeroni F, Pham CG, Kuntzen C, et al. (2006) The NF-kappaB-mediated control of the JNK cascade in the antagonism of programmed cell death in health and disease. Cell Death Differ 13: 712-729.
5. Mattson MP, Meffert MK (2006) Roles for NF-kappaB in nerve cell survival, plasticity, and disease. Cell Death Differ 13: 852-860.
6. Roux PP, Blenis J (2004) ERK and p38 MAPK-activated protein kinases: a family of protein kinases with diverse biological functions. Microbiol Mol Biol Rev 68: 320-344.
7. Thomas GM, Huganir RL (2004) MAPK cascade signalling and synaptic plasticity. Nat Rev Neurosci 5: 173-183.
8. Johnson GL, Nakamura K (2007) The c-jun kinase/stress-activated pathway: Regulation, function and role in human disease. Biochim Biophys Acta.
9. Falschlehner C, Emmerich CH, Gerlach B, Walczak H (2007) TRAIL signalling: Decisions between life and death. Int J Biochem Cell Biol.
10. Franke TF, Hornik CP, Segev L, Shostak GA, Sugimoto C (2003) PI3K/Akt and apoptosis: size matters. Oncogene 22: 8983-8998.
11. Brazil DP, Yang ZZ, Hemmings BA (2004) Advances in protein kinase B signalling: AKTion on multiple fronts. Trends Biochem Sci 29: 233-242.
12. El Yazidi-Belkoura I, Adriaenssens E, Dolle L, Descamps S, Hondermarck H (2003) Tumor necrosis factor receptor-associated death domain protein is involved in the neurotrophin receptor-mediated antiapoptotic activity of nerve growth factor in breast cancer cells. J Biol Chem 278: 16952-16956.
13. Descamps S, Toillon RA, Adriaenssens E, Pawlowski V, Cool SM, et al. (2001) Nerve growth factor stimulates proliferation and survival of human breast cancer cells through two distinct signaling pathways. J Biol Chem 276: 17864-17870.
14. Gainetdinov RR, Fumagalli F, Wang YM, Jones SR, Levey AI, et al. (1998) Increased MPTP neurotoxicity in vesicular monoamine transporter 2 heterozygote knockout mice. J Neurochem 70: 1973-1978.
15. Imai Y, Kimura T, Murakami A, Yajima N, Sakamaki K, et al. (1999) The CED-4-homologous protein FLASH is involved in Fas-mediated activation of caspase-8 during apoptosis. Nature 398: 777-785.
16. Jun JI, Chung CW, Lee HJ, Pyo JO, Lee KN, et al. (2005) Role of FLASH in caspase-8-mediated activation of NF-kappaB: dominant-negative function of FLASH mutant in NF-kappaB signaling pathway. Oncogene 24: 688-696.
17. Gervais FG, Singaraja R, Xanthoudakis S, Gutekunst CA, Leavitt BR, et al. (2002) Recruitment and activation of caspase-8 by the Huntingtin-interacting protein Hip-1 and a novel partner Hippi. Nat Cell Biol 4: 95-105.
18. Pype S, Declercq W, Ibrahimi A, Michiels C, Van Rietschoten JG, et al. (2000) TTRAP, a novel protein that associates with CD40, tumor necrosis factor (TNF) receptor-75 and TNF receptor-associated factors (TRAFs), and that inhibits nuclear factor-kappa B activation. J Biol Chem 275: 18586-18593.
19. Bonif M, Meuwis MA, Close P, Benoit V, Heyninck K, et al. (2006) TNFalpha- and IKKbeta-mediated TANK/I-TRAF phosphorylation: implications for interaction with NEMO/IKKgamma and NF-kappaB activation. Biochem J 394: 593-603.
20. Chan SL, Tan KO, Zhang L, Yee KS, Ronca F, et al. (1999) F1Aalpha, a death receptor-binding protein homologous to the Caenorhabditis elegans sex-determining protein, FEM-1, is a caspase substrate that mediates apoptosis. J Biol Chem 274: 32461-32468.
21. Liu TC, Huang CJ, Chu YC, Wei CC, Chou CC, et al. (2000) Cloning and expression of ZAK, a mixed lineage kinase-like protein containing a leucine-zipper and a sterile-alpha motif. Biochem Biophys Res Commun 274: 811-816.
22. Tosti E, Waldbaum L, Warshaw G, Gross EA, Ruggieri R (2004) The stress kinase MRK contributes to regulation of DNA damage checkpoints through a p38gamma-independent pathway. J Biol Chem 279: 47652-47660.
23. Cho S, Ko HM, Kim JM, Lee JA, Park JE, et al. (2004) Positive regulation of apoptosis signal-regulating kinase 1 by hD53L1. J Biol Chem 279: 16050-16056.
24. Roussigne M, Cayrol C, Clouaire T, Amalric F, Girard JP (2003) THAP1 is a nuclear proapoptotic factor that links prostate-apoptosis-response-4 (Par-4) to PML nuclear bodies. Oncogene 22: 2432-2442.
25. Nahta R, Yuan LX, Fiterman DJ, Zhang L, Symmans WF, et al. (2006) B cell translocation gene 1 contributes to antisense Bcl-2-mediated apoptosis in breast cancer cells. Mol Cancer Ther 5: 1593-1601.
26. Yousefi S, Perozzo R, Schmid I, Ziemiecki A, Schaffner T, et al. (2006) Calpain-mediated cleavage of Atg5 switches autophagy to apoptosis. Nat Cell Biol 8: 1124-1132.

27. Sabourin LA, Tamai K, Seale P, Wagner J, Rudnicki MA (2000) Caspase 3 cleavage of the Ste20-related kinase SLK releases and activates an apoptosis-inducing kinase domain and an actin-disassembling region. Mol Cell Biol 20: 684-696.
28. Hao W, Takano T, Guillemette J, Papillon J, Ren G, et al. (2006) Induction of apoptosis by the Ste20-like kinase SLK, a germinal center kinase that activates apoptosis signal-regulating kinase and p38. J Biol Chem 281: 3075-3084.
29. Samantaray S, Knaryan VH, Guyton MK, Matzelle DD, Ray SK, et al. (2007) The parkinsonian neurotoxin rotenone activates calpain and caspase-3 leading to motoneuron degeneration in spinal cord of Lewis rats. Neuroscience 146: 741-755.
30. Chen MJ, Yap YW, Choy MS, Koh CH, Seet SJ, et al. (2006) Early induction of calpains in rotenone-mediated neuronal apoptosis. Neurosci Lett 397: 69-73.
31. Derouet-Humbert E, Roemer K, Bureik M (2005) Adrenodoxin (Adx) and CYP11A1 (P450scc) induce apoptosis by the generation of reactive oxygen species in mitochondria. Biol Chem 386: 453-461.
32. Relaix F, Wei XJ, Wu X, Sassoon DA (1998) Peg3/Pw1 is an imprinted gene involved in the TNF-NFkappaB signal transduction pathway. Nat Genet 18: 287-291.
33. Johnson MD, Wu X, Aithmitti N, Morrison RS (2002) Peg3/Pw1 is a mediator between p53 and Bax in DNA damage-induced neuronal death. J Biol Chem 277: 23000-23007.
34. Ham J, Towers E, Gilley J, Terzano S, Randall R (2005) BH3-only proteins: key regulators of neuronal apoptosis. Cell Death Differ 12: 1015-1020.
35. Kim H, Rafiuddin-Shah M, Tu HC, Jeffers JR, Zambetti GP, et al. (2006) Hierarchical regulation of mitochondrion-dependent apoptosis by BCL-2 subfamilies. Nat Cell Biol 8: 1348-1358.
36. Shibue T, Taniguchi T (2006) BH3-only proteins: integrated control point of apoptosis. Int J Cancer 119: 2036-2043.
37. Akhtar RS, Geng Y, Klocke BJ, Latham CB, Villunger A, et al. (2006) BH3-only proapoptotic Bcl-2 family members Noxa and Puma mediate neural precursor cell death. J Neurosci 26: 7257-7264.
38. Akhtar RS, Geng Y, Klocke BJ, Roth KA (2006) Neural precursor cells possess multiple p53-dependent apoptotic pathways. Cell Death Differ 13: 1727-1739.
39. Kiryu-Seo S, Hirayama T, Kato R, Kiyama H (2005) Noxa is a critical mediator of p53-dependent motor neuron death after nerve injury in adult mouse. J Neurosci 25: 1442-1447.
40. Li J, Lee B, Lee AS (2006) Endoplasmic reticulum stress-induced apoptosis: multiple pathways and activation of p53-up-regulated modulator of apoptosis (PUMA) and NOXA by p53. J Biol Chem 281: 7260-7270.
41. Ihrie RA, Attardi LD (2004) Perp-etrating p53-dependent apoptosis. Cell Cycle 3: 267-269.
42. Roos WP, Kaina B (2006) DNA damage-induced cell death by apoptosis. Trends Mol Med 12: 440-450.
43. Fujiuchi N, Aglipay JA, Ohtsuka T, Maehara N, Sahin F, et al. (2004) Requirement of IFI16 for the maximal activation of p53 induced by ionizing radiation. J Biol Chem 279: 20339-20344.
44. Phan RT, Dalla-Favera R (2004) The BCL6 proto-oncogene suppresses p53 expression in germinal-centre B cells. Nature 432: 635-639.
45. Iyer NG, Chin SF, Ozdag H, Daigo Y, Hu DE, et al. (2004) p300 regulates p53-dependent apoptosis after DNA damage in colorectal cancer cells by modulation of PUMA/p21 levels. Proc Natl Acad Sci U S A 101: 7386-7391.
46. Inukai T, Inoue A, Kurosawa H, Goi K, Shinjyo T, et al. (1999) SLUG, a ces-1-related zinc finger transcription factor gene with antiapoptotic activity, is a downstream target of the E2A-HLF oncoprotein. Mol Cell 4: 343-352.
47. Tribulo C, Aybar MJ, Sanchez SS, Mayor R (2004) A balance between the anti-apoptotic activity of Slug and the apoptotic activity of msx1 is required for the proper development of the neural crest. Dev Biol 275: 325-342.
48. Park K, Kim K, Rho SB, Choi K, Kim D, et al. (2005) Homeobox Msx1 interacts with p53 tumor suppressor and inhibits tumor growth by inducing apoptosis. Cancer Res 65: 749-757.
49. Liang XQ, Cao EH, Zhang Y, Qin JF (2004) A P53 target gene, PIG11, contributes to chemosensitivity of cells to arsenic trioxide. FEBS Lett 569: 94-98.
50. Morin PJ, Vogelstein B, Kinzler KW (1996) Apoptosis and APC in colorectal tumorigenesis. Proc Natl Acad Sci U S A 93: 7950-7954.
51. Renard CA, Labalette C, Armengol C, Cougot D, Wei Y, et al. (2007) Tbx3 is a downstream target of the Wnt/beta-catenin pathway and a critical mediator of beta-catenin survival functions in liver cancer. Cancer Res 67: 901-910.
52. Houseweart MK, Pennacchio LA, Vilaythong A, Peters C, Noebels JL, et al. (2003) Cathepsin B but not cathepsins L or S contributes to the pathogenesis of Unverricht-Lundborg progressive myoclonus epilepsy (EPM1). J Neurobiol 56: 315-327.
53. Felbor U, Kessler B, Mothes W, Goebel HH, Ploegh HL, et al. (2002) Neuronal loss and brain atrophy in mice lacking cathepsins B and L. Proc Natl Acad Sci U S A 99: 7883-7888.
54. Oppenheim RW, Houenou LJ, Parsadanian AS, Prevette D, Snider WD, et al. (2000) Glial cell line-derived neurotrophic factor and developing mammalian motoneurons: regulation of programmed cell death among motoneuron subtypes. J Neurosci 20: 5001-5011.
55. Marco S, Saura J, Perez-Navarro E, Jose Marti M, Tolosa E, et al. (2002) Regulation of c-Ret, GFRalpha1, and GFRalpha2 in the substantia nigra pars compacta in a rat model of Parkinson's disease. J Neurobiol 52: 343-351.
56. Buttini M, Orth M, Bellosta S, Akeefe H, Pitas RE, et al. (1999) Expression of human apolipoprotein E3 or E4 in the brains of Apoe-/- mice: isoform-specific effects on neurodegeneration. J Neurosci 19: 4867-4880.
57. Mahley RW, Weisgraber KH, Huang Y (2006) Apolipoprotein E4: a causative factor and therapeutic target in neuropathology, including Alzheimer's disease. Proc Natl Acad Sci U S A 103: 5644-5651.
58. Veinbergs I, Everson A, Sagara Y, Masliah E (2002) Neurotoxic effects of apolipoprotein E4 are mediated via dysregulation of calcium homeostasis. J Neurosci Res 67: 379-387.
59. Quinn CM, Kagedal K, Terman A, Stroikin U, Brunk UT, et al. (2004) Induction of fibroblast apolipoprotein E expression during apoptosis, starvation-induced growth arrest and mitosis. Biochem J 378: 753-761.
60. Elliott DA, Kim WS, Jans DA, Garner B (2007) Apoptosis induces neuronal apolipoprotein-E synthesis and localization in apoptotic bodies. Neurosci Lett 416: 206-210.

61. Tafolla E, Wang S, Wong B, Leong J, Kapila YL (2005) JNK1 and JNK2 oppositely regulate p53 in signaling linked to apoptosis triggered by an altered fibronectin matrix: JNK links FAK and p53. J Biol Chem 280: 19992-19999.
62. Li L, Ren CH, Tahir SA, Ren C, Thompson TC (2003) Caveolin-1 maintains activated Akt in prostate cancer cells through scaffolding domain binding site interactions with and inhibition of serine/threonine protein phosphatases PP1 and PP2A. Mol Cell Biol 23: 9389-9404.
63. Alon T, Hemo I, Itin A, Pe'er J, Stone J, et al. (1995) Vascular endothelial growth factor acts as a survival factor for newly formed retinal vessels and has implications for retinopathy of prematurity. Nat Med 1: 1024-1028.
64. Gerber HP, Dixit V, Ferrara N (1998) Vascular endothelial growth factor induces expression of the antiapoptotic proteins Bcl-2 and A1 in vascular endothelial cells. J Biol Chem 273: 13313-13316.
65. Tahara E, Jr., Tahara H, Kanno M, Naka K, Takeda Y, et al. (2005) G1P3, an interferon inducible gene 6-16, is expressed in gastric cancers and inhibits mitochondrial-mediated apoptosis in gastric cancer cell line TMK-1 cell. Cancer Immunol Immunother 54: 729-740.
66. Dhar A, Gardner J, Borgmann K, Wu L, Ghorpade A (2006) Novel role of TGF-beta in differential astrocyte-TIMP-1 regulation: implications for HIV-1-dementia and neuroinflammation. J Neurosci Res 83: 1271-1280.
67. Liu XW, Taube ME, Jung KK, Dong Z, Lee YJ, et al. (2005) Tissue inhibitor of metalloproteinase-1 protects human breast epithelial cells from extrinsic cell death: a potential oncogenic activity of tissue inhibitor of metalloproteinase-1. Cancer Res 65: 898-906.
68. Schipper HM (2004) Heme oxygenase expression in human central nervous system disorders. Free Radic Biol Med 37: 1995-2011.
69. Durante W (2003) Heme oxygenase-1 in growth control and its clinical application to vascular disease. J Cell Physiol 195: 373-382.
70. Song W, Su H, Song S, Paudel HK, Schipper HM (2006) Over-expression of heme oxygenase-1 promotes oxidative mitochondrial damage in rat astroglia. J Cell Physiol 206: 655-663.
71. Thom SR, Fisher D, Xu YA, Notarfrancesco K, Ischiropoulos H (2000) Adaptive responses and apoptosis in endothelial cells exposed to carbon monoxide. Proc Natl Acad Sci U S A 97: :1305–1310.
72. Sherer TB, Betarbet R, Stout AK, Lund S, Baptista M, et al. (2002) An in vitro model of Parkinson's disease: linking mitochondrial impairment to altered alpha-synuclein metabolism and oxidative damage. J Neurosci 22: 7006-7015.
73. Kaur D, Andersen J (2004) Does cellular iron dysregulation play a causative role in Parkinson's disease? Ageing Res Rev 3: 327-343.
74. Lee DW, Andersen JK, Kaur D (2006) Iron dysregulation and neurodegeneration: the molecular connection. Mol Interv 6: 89-97.
75. Ryter SW, Tyrrell RM (2000) The heme synthesis and degradation pathways: role in oxidant sensitivity. Heme oxygenase has both pro- and antioxidant properties. Free Radic Biol Med 28: 289-309.
76. Pereira PJ, Macedo-Ribeiro S, Parraga A, Perez-Luque R, Cunningham O, et al. (2001) Structure of human biliverdin IXbeta reductase, an early fetal bilirubin IXbeta producing enzyme. Nat Struct Biol 8: 215-220.
77. Puccetti P (2007) On watching the watchers: IDO and type I/II IFN. Eur J Immunol 37: 876-879.
78. Hill M, Pereira V, Chauveau C, Zagani R, Remy S, et al. (2005) Heme oxygenase-1 inhibits rat and human breast cancer cell proliferation: mutual cross inhibition with indoleamine 2,3-dioxygenase. Faseb J 19: 1957-1968.
79. Bubici C, Papa S, Dean K, Franzoso G (2006) Mutual cross-talk between reactive oxygen species and nuclear factor-kappa B: molecular basis and biological significance. Oncogene 25: 6731-6748.
80. Pham CG, Bubici C, Zazzeroni F, Papa S, Jones J, et al. (2004) Ferritin heavy chain upregulation by NF-kappaB inhibits TNFalpha-induced apoptosis by suppressing reactive oxygen species. Cell 119: 529-542.
81. Pham CG, Bubici C, Zazzeroni F, Knabb JR, Papa S, et al. (2007) Upregulation of Twist-1 by NF-{kappa}B Blocks Cytotoxicity Induced by Chemotherapeutic Drugs. Mol Cell Biol 27: 3920-3935.
82. Molina-Jimenez MF, Sanchez-Reus MI, Cascales M, Andres D, Benedi J (2005) Effect of fraxetin on antioxidant defense and stress proteins in human neuroblastoma cell model of rotenone neurotoxicity. Comparative study with myricetin and N-acetylcysteine. Toxicol Appl Pharmacol 209: 214-225.
83. Manna SK, Kuo MT, Aggarwal BB (1999) Overexpression of gamma-glutamylcysteine synthetase suppresses tumor necrosis factor-induced apoptosis and activation of nuclear transcription factor-kappa B and activator protein-1. Oncogene 18: 4371-4382.
84. Botta D, Franklin CC, White CC, Krejsa CM, Dabrowski MJ, et al. (2004) Glutamate-cysteine ligase attenuates TNF-induced mitochondrial injury and apoptosis. Free Radic Biol Med 37: 632-642.
85. Zhu M, Qin ZJ, Hu D, Munishkina LA, Fink AL (2006) Alpha-synuclein can function as an antioxidant preventing oxidation of unsaturated lipid in vesicles. Biochemistry 45: 8135-8142.
86. Cabin DE, Shimazu K, Murphy D, Cole NB, Gottschalk W, et al. (2002) Synaptic vesicle depletion correlates with attenuated synaptic responses to prolonged repetitive stimulation in mice lacking alpha-synuclein. J Neurosci 22: 8797-8807.
87. Yavich L, Tanila H, Vepsalainen S, Jakala P (2004) Role of alpha-synuclein in presynaptic dopamine recruitment. J Neurosci 24: 11165-11170.
88. Lee M, Hyun D, Halliwell B, Jenner P (2001) Effect of the overexpression of wild-type or mutant alpha-synuclein on cell susceptibility to insult. J Neurochem 76: 998-1009.
89. Manning-Bog AB, McCormack AL, Purisai MG, Bolin LM, Di Monte DA (2003) Alpha-synuclein overexpression protects against paraquat-induced neurodegeneration. J Neurosci 23: 3095-3099.
90. Seo JH, Rah JC, Choi SH, Shin JK, Min K, et al. (2002) Alpha-synuclein regulates neuronal survival via Bcl-2 family expression and PI3/Akt kinase pathway. Faseb J 16: 1826-1828.
91. Sidhu A, Wersinger C, Moussa CE, Vernier P (2004) The Role of (alpha)-Synuclein in Both Neuroprotection and Neurodegeneration. Ann N Y Acad Sci 1035: 250-270.
92. Alves da Costa C, Dunys J, Brau F, Wilk S, Cappai R, et al. (2006) 6-Hydroxydopamine but not 1-methyl-4-phenylpyridinium abolishes alpha-synuclein anti-apoptotic phenotype by inhibiting its proteasomal degradation and by promoting its aggregation. J Biol Chem 281: 9824-9831.
93. Hashimoto M, Hsu LJ, Rockenstein E, Takenouchi T, Mallory M, et al. (2002) alpha-Synuclein protects against oxidative stress via inactivation of the c-Jun N-terminal kinase stress-signaling pathway in neuronal cells. J Biol Chem 277: 11465-11472.
94. Iwata A, Maruyama M, Kanazawa I, Nukina N (2001) alpha-Synuclein affects the MAPK pathway and accelerates cell death. J Biol Chem 276: 45320-45329.

95. Kholodilov NG, Oo TF, Burke RE (1999) Synuclein expression is decreased in rat substantia nigra following induction of apoptosis by intrastriatal 6-hydroxydopamine. Neurosci Lett 275: 105-108.
96. Betarbet R, Sherer TB, MacKenzie G, Garcia-Osuna M, Panov AV, et al. (2000) Chronic systemic pesticide exposure reproduces features of Parkinson's disease. Nat Neurosci 3: 1301-1306.
97. Betarbet R, Canet-Aviles RM, Sherer TB, Mastroberardino PG, McLendon C, et al. (2006) Intersecting pathways to neurodegeneration in Parkinson's disease: effects of the pesticide rotenone on DJ-1, alpha-synuclein, and the ubiquitin-proteasome system. Neurobiol Dis 22: 404-420.
98. Vila M, Vukosavic S, Jackson-Lewis V, Neystat M, Jakowec M, et al. (2000) Alpha-synuclein up-regulation in substantia nigra dopaminergic neurons following administration of the parkinsonian toxin MPTP. J Neurochem 74: 721-729.
99. Lee HG, Zhu X, Takeda A, Perry G, Smith MA (2006) Emerging evidence for the neuroprotective role of alpha-synuclein. Exp Neurol 200: 1-7.
100. Sanjo H, Takeda K, Tsujimura T, Ninomiya-Tsuji J, Matsumoto K, et al. (2003) TAB2 is essential for prevention of apoptosis in fetal liver but not for interleukin-1 signaling. Mol Cell Biol 23: 1231-1238.
101. Nishina H, Vaz C, Billia P, Nghiem M, Sasaki T, et al. (1999) Defective liver formation and liver cell apoptosis in mice lacking the stress signaling kinase SEK1/MKK4. Development 126: 505-516.
102. Gan B, Peng X, Nagy T, Alcaraz A, Gu H, et al. (2006) Role of FIP200 in cardiac and liver development and its regulation of TNFalpha and TSC-mTOR signaling pathways. J Cell Biol 175: 121-133.
103. Li S, Perlman DM, Peterson MS, Burrichter D, Avdulov S, et al. (2004) Translation initiation factor 4E blocks endoplasmic reticulum-mediated apoptosis. J Biol Chem 279: 21312-21317.
104. Humbert S, Saudou F (2005) [Huntington's disease: intracellular signaling pathways and neuronal death]. J Soc Biol 199: 247-251.
105. Gabai VL, Yaglom JA, Volloch V, Meriin AB, Force T, et al. (2000) Hsp72-mediated suppression of c-Jun N-terminal kinase is implicated in development of tolerance to caspase-independent cell death. Mol Cell Biol 20: 6826-6836.
106. Clemons NJ, Buzzard K, Steel R, Anderson RL (2005) Hsp72 inhibits Fas-mediated apoptosis upstream of the mitochondria in type II cells. J Biol Chem 280: 9005-9012.
107. Wingrave JM, Sribnick EA, Wilford GG, Matzelle DD, Mou JA, et al. (2004) Higher calpastatin levels correlate with resistance to calpain-mediated proteolysis and neuronal apoptosis in juvenile rats after spinal cord injury. J Neurotrauma 21: 1240-1254.
108. Porn-Ares MI, Samali A, Orrenius S (1998) Cleavage of the calpain inhibitor, calpastatin, during apoptosis. Cell Death Differ 5: 1028-1033.
109. Kim KA, Lee YA, Shin MH (2007) Calpain-dependent calpastatin cleavage regulates caspase-3 activation during apoptosis of Jurkat T cells induced by Entamoeba histolytica. Int J Parasitol.
110. Bleckmann SC, Blendy JA, Rudolph D, Monaghan AP, Schmid W, et al. (2002) Activating transcription factor 1 and CREB are important for cell survival during early mouse development. Mol Cell Biol 22: 1919-1925.
111. Jean D, Harbison M, McConkey DJ, Ronai Z, Bar-Eli M (1998) CREB and its associated proteins act as survival factors for human melanoma cells. J Biol Chem 273: 24884-24890.
112. Aggarwal BB (2003) Signalling pathways of the TNF superfamily: a double-edged sword. Nat Rev Immunol 3: 745-756.
113. Walczak H, Degli-Esposti MA, Johnson RS, Smolak PJ, Waugh JY, et al. (1997) TRAIL-R2: a novel apoptosis-mediating receptor for TRAIL. Embo J 16: 5386-5397.
114. Fernandez-Fernandez MR, Veprintsev DB, Fersht AR (2005) Proteins of the S100 family regulate the oligomerization of p53 tumor suppressor. Proc Natl Acad Sci U S A 102: 4735-4740.
115. Kogel D, Peters M, Konig HG, Hashemi SM, Bui NT, et al. (2004) S100B potently activates p65/c-Rel transcriptional complexes in hippocampal neurons: Clinical implications for the role of S100B in excitotoxic brain injury. Neuroscience 127: 913-920.
116. Wainwright MS, Craft JM, Griffin WS, Marks A, Pineda J, et al. (2004) Increased susceptibility of S100B transgenic mice to perinatal hypoxia-ischemia. Ann Neurol 56: 61-67.
117. Vilar M, Murillo-Carretero M, Mira H, Magnusson K, Besset V, et al. (2006) Bex1, a novel interactor of the p75 neurotrophin receptor, links neurotrophin signaling to the cell cycle. Embo J 25: 1219-1230.
118. Tchilian EZ, Beverley PC (2006) Altered CD45 expression and disease. Trends Immunol 27: 146-153.
119. Sakuta H, Inaba K, Muramatsu S (1996) Calcitonin gene-related peptide enhances apoptosis of thymocytes. J Neuroimmunol 67: 103-109.
120. Millet I, Phillips RJ, Sherwin RS, Ghosh S, Voll RE, et al. (2000) Inhibition of NF-kappaB activity and enhancement of apoptosis by the neuropeptide calcitonin gene-related peptide. J Biol Chem 275: 15114-15121.
