# Supplementary material for: Transcriptome Analysis of a Rotenone Model of Parkinsonism Reveals Complex I-Tied and -Untied Toxicity Mechanisms Common to Neurodegenerative Diseases
Source: PLoS One. 2012 Sep 7;7(9):e44700. doi: 10.1371/journal.pone.0044700 (PMC3436760; doi:10.1371/journal.pone.0044700)
Supplement: Figure S2 — Expression patterns of 58 common differentially-regulated genes (DRGs) by 5 nM rotenone at 4 weeks. Expression pattern of three transcriptome analysis experiments of SK-N-MC cells chronically treated with 5 nM rotenone or vehicle (0 nM) for 4 weeks (4w). 58 genes of the 898 DRGs detected by dCHIP in Greene et al., [29], data were also differentially-regulated in our 4w5 nM treatment group, as shown in (A); for comparison the expression pattern of the same DRGs in Greene et al., [29], data is shown in (B), where GSM107862, GSM107863, and GSM107864 correspond to the vehicle-treated samples at 4 weeks and GSM107865, GSM107866, and GSM107867 correspond to the 5 nM rotenone treated samples at 4 weeks. DRGs were clustered by hierarchical average-linkage analysis, as implemented in the MeV software accessible in the TM4 suite [118], and shown in colorgrams depicting the expression level of the genes (rows) in each individual sample (columns). Expression above the mean is displayed in red and below the mean in blue (for normalized scale see bar on top). In (C) below, similar clustering analysis of the 58 commonly affected DRGs was applied to the same samples from both studies in order to visualize the distance (blue shade in dendogram) between control and treated samples in both datasets, and the differences in magnitude and direction of changes between datasets under the same scale (top). Format: PDF Size: 509 KB; This file can be viewed with: Adobe Acrobat Reader. (PDF) [file pone.0044700.s002.pdf]

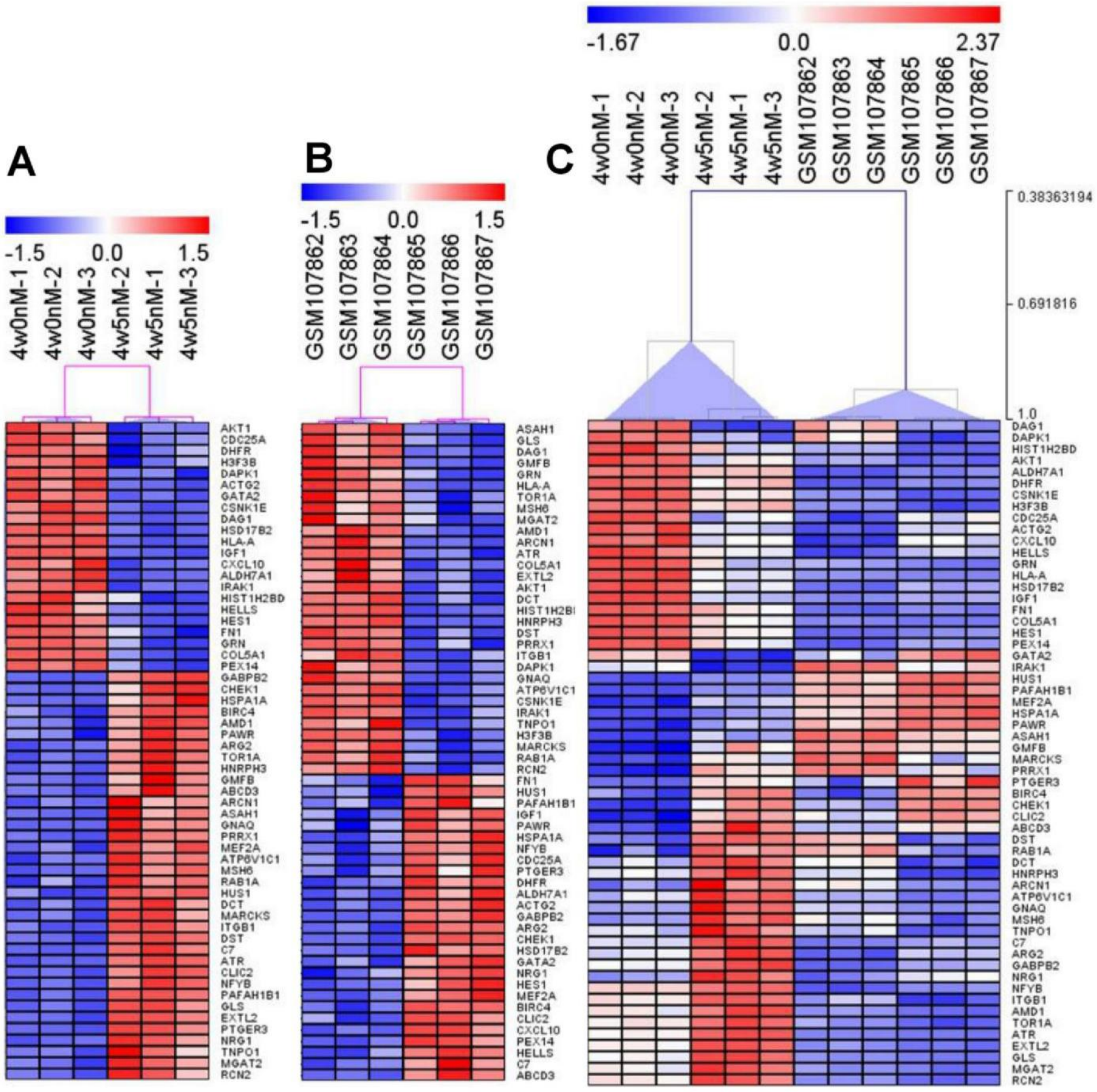

Figure S2. Expression patterns of 58 common differentially-regulated genes (DRGs) by 5 nM rotenone at 4 weeks. Expression pattern of three transcriptome analysis experiments of SK-N-MC cells chronically treated with 5 nM rotenone or vehicle (0 nM) for 4 weeks (4w). 58 genes of the 898 DRGs detected by dCHIP in Greene et al., [29], data were also differentially-regulated in our 4w5nM treatment group, as shown in (**A**); for comparison the expression pattern of the same DRGs in Greene et al., [29], data is shown in (**B**), where GSM107862, GSM107863, and GSM107864 correspond to the vehicle-treated samples at 4 weeks and GSM107865, GSM107866, and GSM107867 correspond to the 5 nM rotenone treated samples at 4 weeks. DRGs were clustered by hierarchical average-linkage analysis, as implemented in the MeV software accessible in the TM4 suite [132], and shown in colorgrams depicting the expression level of the genes (rows) in each individual sample (columns). Expression above the mean is displayed in red and below the mean in blue (for normalized scale see bar on top). In (**C**) below, similar clustering analysis of the 58 commonly affected DRGs was applied to the same samples from both studies in order to visualize the distance (blue shade in dendogram) between control and treated samples in both datasets, and the differences in magnitude and direction of changes between datasets under the same scale (top).
