# Supplementary material for: Transcriptome Analysis of a Rotenone Model of Parkinsonism Reveals Complex I-Tied and -Untied Toxicity Mechanisms Common to Neurodegenerative Diseases
Source: PLoS One. 2012 Sep 7;7(9):e44700. doi: 10.1371/journal.pone.0044700 (PMC3436760; doi:10.1371/journal.pone.0044700)
Supplement: Figure S3 — Proliferation kinetics curves. Cell proliferation curves for populations of rotenone-treated and vehicle-treated cells during exponential growth for a 30 days period of culture. SK-N-MC cells were seeded at a density of ∼3×10^3^/cm2 and grown, in the same medium and conditions as for the transcriptome analysis, to a confluence not higher than ∼70% in the absence, and presence of 5 nM and 50 nM rotenone. Data are presented as mean ± S.E.M (N = 3) of the number of cells at each time point; plotted in linear scale (A) and in logarithmic scale (B). The exponential curve fitting in (A), which assumes that that all cells are actively dividing to give rise to two daughter cells, was performed with the Prism 5.0 software (Graphpad Inc.), using the growth equation : Nt = N0×2t/DT where N0 is the initial number of cells, Nt is the number of cells at time, t, and DT is the division time; and used to determine the growth rate constant, which was subsequently used to ascertain the effects of rotenone on growth rate ( Fig. 1A ), the doubling times ( Fig. 1B ), and the fraction of dividing cells ( Fig. 1C ). Format: PDF Size: 261 KB; This file can be viewed with: Adobe Acrobat Reader. (PDF) [file pone.0044700.s003.pdf]

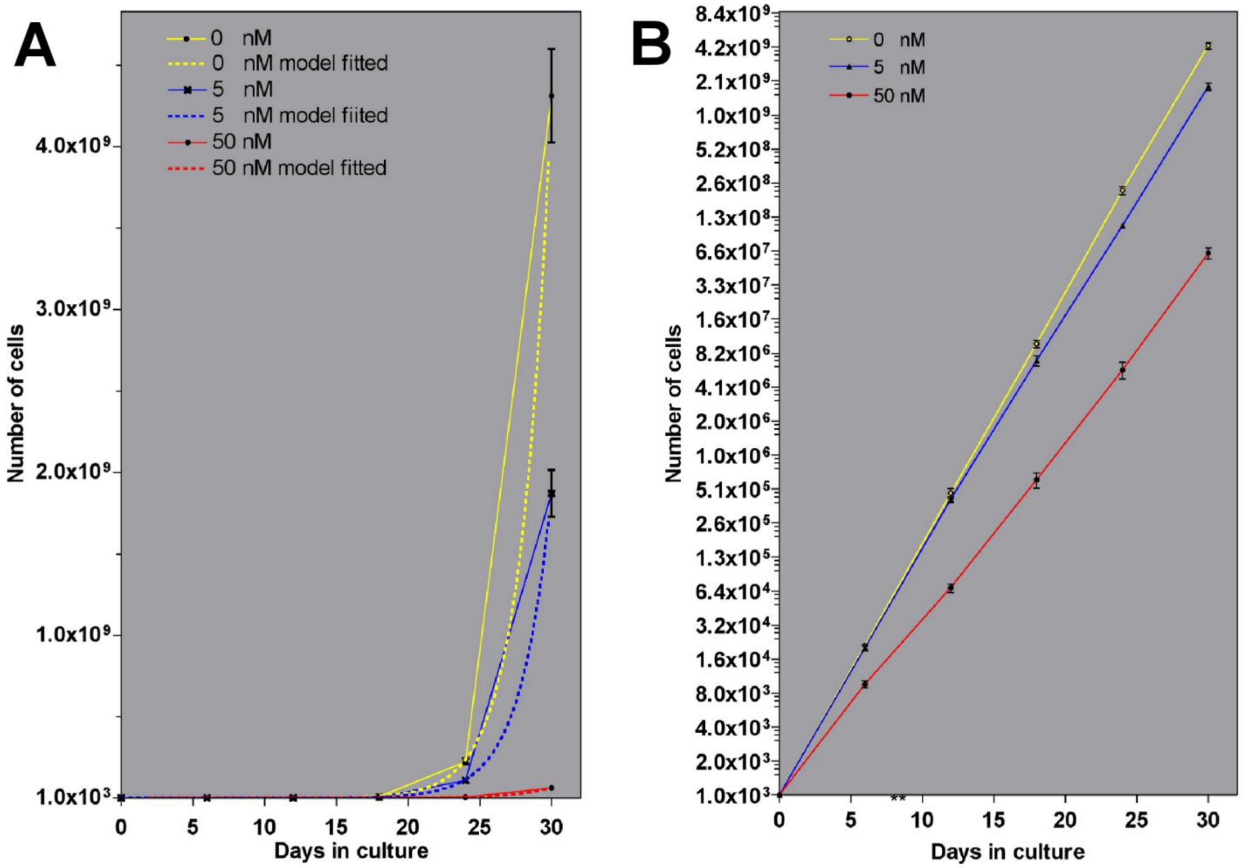

Figure S3. Proliferation kinetics curves: Cell proliferation curves for populations of rotenone-treated and vehicle-treated cells during exponential growth for a 30 days period of culture. Data are presented as mean ± S.E.M (N = 3) of the number of cells at each time point; plotted in linear scale (**A**) and in logarithmic scale (**B**). The exponential curve fitting in (A), which assumes that that all cells are actively dividing to give rise to two daughter cells, was performed with the Prism 5.0 software (Graphpad Inc.), using the growth equation : $Nt = N_0 x 2^{t/DT}$ where N_0 is the initial number of cells, Nt is the number of cells at time, t, and DT is the division time; and used to determine the growth rate constant, which was subsequently used to ascertain the effects of rotenone on growth rate (**Fig. 1A),** the doubling times (**Fig. 1B**), and the fraction of dividing cells (**Fig. 1C**).

Assaying rotenone effects on SK-N-MC cells proliferation and cytotoxicity

To assay cell proliferation kinetics, cell population doubling time, fraction of dividing cells, and percentage of dead cells after exposure of the SK-N-MC cell line to rotenone, cells were seeded at a density of ~ $3x10^3/cm^2$ and grown in the same medium and conditions, as the transcriptome analysis, to a confluence not higher than ~70 % in the absence (vehicle only) and presence of 5 nM or 50 nM rotenone. Three wells of plated cells per each condition were trypsinized after 6 days and total cell number was counted on a Neubauer hemocytometer to calculate the mean ± standard deviation (SD). For subsequent time points, every six day for 30 days, the whole cell populations or dilutions, as needed, were transferred to plates with progressively larger surface areas to maintain the initial seeding density (~ $3000/cm^2$) and counted as above. Cell number was graphed to obtain the growth curves and exponential growth curve fitting was performed using the Prism 5.0 software (Graphpad Inc.) (**Fig. S3A**). The growth rate constant under each treatment condition was used to determine growth rates percentages relative to the untreated cell populations (assumed to grow at 100 % rate) (Fig. 6A). The doubling time in the exponential growth phase was calculated , at each time point, for each cell population treatment condition (**Fig. 1B**), using the formula: $PDT = \Delta t \times [\ln 2/(\ln N_t - \ln N_0)]$, which assumes that all cells are actively dividing, and where PDT is the cell population doubling time, Δt is the duration of the cell growth at each time (for 6 Days; 144 h), N_0 and N_t are the number of cells at the beginning of the experiment and after 144 h, respectively. The dividing or mitotic fraction, at each time point, was estimated (**Fig. 1C**) using the formula for population doubling time (PDT) derived by Deasy et al., (2003), [35]: $PDT = DT \times [\ln(6\alpha-2)/\ln(2\alpha) - 1]$, where DT is the doubling time for the untreated cells (average of all time points = 32.8 h), PDT is the population doubling time calculated at each time point as above, and α is the dividing fraction. The cytotoxicity of rotenone was assayed by determining the percentage of dead cells under each treatment condition, after carefully collecting all detached cells and combining them with the rest of the trypsinized cells prior to counting the cells with using the trypan blue dye exclusion method (**Fig. 1D**).
