# Supplementary material for: Transcriptome Analysis of a Rotenone Model of Parkinsonism Reveals Complex I-Tied and -Untied Toxicity Mechanisms Common to Neurodegenerative Diseases
Source: PLoS One. 2012 Sep 7;7(9):e44700. doi: 10.1371/journal.pone.0044700 (PMC3436760; doi:10.1371/journal.pone.0044700)
Supplement: Table S1 — Microarray data quality control and differentially-regulated genes (DRGs). Format. PDF Size: 306 KB; This file can be viewed with: Adobe Acrobat Reader. (PDF) [file pone.0044700.s004.pdf]

Table S1. Array data quality control and rotenone differentially-regulated genes (DRGs)

**treatment**		**array data quality control**			**number of DRGs** [c, d]		
time (**weeks**)	rotenone (**nM**)	% present calls[a,b] (mean ± sd)	median intensity[a,b] (mean ± sd)	% single outliers (mean ± sd)	up	down	total
1	**0**	59 ± 3	185 ± 33	0.1 ± 0.02	0	0	0
	**5**	61 ± 2	177 ± 18	0.1 ± 0.03	59	16	75
	**50**	60 ± 5	155 ± 9	0.1 ± 0.03	64	48	112
			mutually affected by both doses →		45	8	53
4	**0**	62 ± 1	199 ± 17	0.1 ± 0.02	0	0	0
	**5**	63 ± 1	270 ± 33	0.1 ± 0.02	288	169	457
	**50**	62 ± 0	249 ± 6	0.1 ± 0.01	379	240	619
			mutually affected by both doses →		145	106	251

Abbreviations: DRG: differentially-regulated gene. **Notes: a**: by dCHIP; **b**:not significant (p<0.05) difference; **c:** by dCHIP with criteria: fold change >2, p<0.05; **d:** significance analysis of microarray, multiple test correction, FDR (%) 90th % tile <1% D > 1.2
