# Supplementary material for: Transcriptome Analysis of a Rotenone Model of Parkinsonism Reveals Complex I-Tied and -Untied Toxicity Mechanisms Common to Neurodegenerative Diseases
Source: PLoS One. 2012 Sep 7;7(9):e44700. doi: 10.1371/journal.pone.0044700 (PMC3436760; doi:10.1371/journal.pone.0044700)
Supplement: Table S3 — Rotenone-regulated genes associated to neurological and vascular diseases. Format: PDF Size: 438 KB; This file can be viewed with: Adobe Acrobat Reader. (PDF) [file pone.0044700.s006.pdf]

Table S3. Rotenone-regulated genes associated to neurological and vascular diseases

entrez ID	gene symbol	gene description	fold c. (4 weeks)		associated diseases[1]
			5 nM	50 nM	
5788	PTPRC	protein tyrosine phosphatase, receptor type, C	**3.5**	**-4.5**	AD, MS
3303	HSPA1A	heat shock 70kDa protein 1A	**3.3**	**-3.0**	AD
6622	SNCA	synuclein, alpha	**2.6**	**2.0**	AD, PD, Db
3480	IGF1R	insulin-like growth factor 1 receptor	**2.5**	**2.1**	ND, Db
831	CAST	calpastatin		**2.3**	PD
102	ADAM10	a disintegrin and metalloproteinase domain 10	**2.4**	**2.5**	AD[2]
590	BCHE	butyrylcholinesterase		**2.5**	AD, Db
6934	TCF7L2:	transcription factor 7-like 2 (T-cell specific)		**2.5**	Db
7019	TFAM	transcription factor A, mitochondrial	**2.3**	**2.4**	AD
8754	ADAM9	ADAM metallopeptidase domain 9	**2.2**		AD[2]
6648	SOD2	superoxide dismutase 2, mitochondrial	**2.1**	**2.8**	AD
8301	PICALM	phosphatidylinositol binding clathrin assembly protein	**2.0**	**2.7**	AD[2]
2052	EPHX1	epoxide hydrolase 1, microsomal (xenobiotic)		**-2.2**	ND, AD
2902	GRIN1	glutamate receptor, ionotropic, N-methyl D-aspartate 1		**-2.3**	ND, AD
356	FASLG	Fas ligand (TNF superfamily, member 6)	**-2.7**	**-2.6**	AD
2896	GRN	granulin	**-2.1**	**-2.7**	FTLD
323	APBB2	amyloid β (A4) precursor protein bind. fam. B, memb.	**2.6**	**-2.5**	AD
2524	FUT2	fucosyltransferase 2 (secretor status included)	**-2.4**	**-2.6**	ND
6609	SMPD1	sphingomyelin phosphodiesterase 1, acid lysosomal	**-2.8**	**-8.2**	NPD
4318	MMP9	matrix metallopeptidase 9	**-3.0**	**-2.4**	AD, MS
2100	ESR2	estrogen receptor 2 (ER beta)	**-3.1**	**-2.9**	ND, AD
322	APBB1	amyloid β (A4) precursor protein-bind., fam. B, memb.		**-3.4**	AD[2]
3107	HLA-C	major histocompatibility complex, class I, C	**-9.0**	**-2.6**	MS
7076	TIMP1	TIMP metallopeptidase inhibitor 1	**-4.5**	**-3.0**	AD
3106	HLA-B	major histocompatibility complex, class I, B	**-5.7**	**-2.5**	AD, MS
348	APOE	apolipoprotein E	**-4.8**	**-2.7**	AD, PD. MS, FTLD
3162	HMOX1	heme oxygenase 1	**-5.3**	**-2.4**	AD
6572	SLC18A3	solute carrier fam. 18 (vesic. acetylcholine), memb. 3	**-6.6**	**-3.5**	AD
3481	IGF2	insulin-like growth factor 2 (somatomedin A)	**-8.4**	**-4.9**	AD
25825	BACE2	beta-site APP-cleaving enzyme 2		**-4.9**	AD[2]
1585	CYP11B2	cytochrome P450, fam. 11, subfam. B, polypeptide 2	**-4.8**	**-6.2**	ND, Db, CV
1191	CLU	clusterin		**-9.1**	AD[2] , PD[2]

Abbreviations: D: disease**; AD**: Alzheimer D; **CV:** cardiovascular; Db: diabetes; D.; **FTLD**: Frontotemporal lobar degeneration; **fold c. :** fold change **HD**:Huntington D; **MS**:Multiple sclerosis; **ND**:Neurological D; **NPD**:Nieman's Pick D; **PD**:Pakinson D; Official gene symbols are used (http://www.ncbi.nlm.nih.gov/gene/

Notes: 1: Neurological & cardiovascular diseases associated with rotenone changed genes: At least two of the diseases associated with rotenone-changed genes, AD and diabetes, share vascular risk factors in their pathogenesis [1], which makes the notion that transcriptional regulation by rotenone interferes with vascularisation interesting, and is supported by findings of vascular damage in the rat brain by rotenone [2]. Genes associated with diabetes include *SNCA,* which inhibits insulin secretion in pancreas β islet cells [3], and when overexpressed in mouse striatum it upregulates *Tcf7l2* [4], a gene linked to risk for diabetes and essential in β-cell function [5], which was also deregulated by rotenone. Neuron development genes were primarily repressed by the lower dose at 4 weeks, which indicates the potential of low levels of rotenone to influence NDs development and is consistent with the positive association of PD with human chronic exposure to rotenone [6]. Notably, three of the rotenone-regulated genes associated to neurological diseases have been linked to either AD or PD, or both. Explicitly, *CLU*, is associated with PD and AD [7, 8]; *PICALM*, is linked to AD [7]; and *SOD2*, is connected to AD [9]. Importantly, rotenone affected genes that regulate processes connected to NDs; in particular to AD, as their proteins are implicated in Aβ processing and clearance, including CLU [7], APOE [10], *BACE2*, *APBB1, APBB2*, *ADAM10* and *ADAM9* [11].
2: all associated diseases according to the genetic association database (GAD); except for those indicated by a 2; which were not in the GAD but were suggested instead in references [7, 8, 11] below

References

1. Casserly I, Topol E (2004) Convergence of atherosclerosis and Alzheimer’s disease: inflammation, cholesterol, and misfolded proteins. Lancet 363: 1139–1146.
2. Radad K, Hassanein K, Moldzio R, Rausch WD (2011) Vascular damage mediates neuronal and non-neuronal pathology following short and long-term rotenone administration in Sprague-Dawley rats. Exp Toxicol Pathol: PMID: 21676605.
3. Geng X, Lou H, Wang J, Li L, Swanson AL, et al. (2011) alpha-Synuclein binds the K(ATP) channel at insulin-secretory granules and inhibits insulin secretion. Am J Physiol Endocrinol Metab 300: E276-286.
4. Cabeza-Arvelaiz Y, Fleming SM, Richter F, Masliah E, Chesselet MF, et al. (2011) Analysis of striatal transcriptome in mice overexpressing human wild-type alpha-synuclein supports synaptic dysfunction and suggests mechanisms of neuroprotection for striatal neurons. Mol Neurodegener 6: 83.
5. van de Bunt M, Gloyn AL (2010) From genetic association to molecular mechanism. Curr Diab Rep 10: 452-466.
6. Tanner CM, Kamel F, Ross GW, Hoppin JA, Goldman SM, et al. (2011) Rotenone, paraquat, and Parkinson's disease. Environ Health Perspect 119: 866-872.
7. Harold D, Abraham R, Hollingworth P, Sims R, Gerrish A, et al. (2009) Genome-wide association study identifies variants at CLU and PICALM associated with Alzheimer's disease, and shows evidence for additional susceptibility genes. Nature Genetics 41: 1088-1109.
8. Gao J, Huang X, Park Y, Hollenbeck A, Chen H (2011) An exploratory study on CLU, CR1 and PICALM and Parkinson disease. PLoS One 6: e24211.
9. Wiener HW, al. e (2007) An SOD2 polymorhism is associated with Alzheimer's disease development. Genes Brain and Development 6: 770-775.
10. DeMattos RB, Cirrito JR, Parsadanian M, May PC, O'Dell MA, et al. (2004) ApoE and Clusterin Cooperatively Suppress Abeta Levels and Deposition. Evidence that ApoE Regulates Extracellular Abeta Metabolism In Vivo. Neuron 41: 193-202.
11. Wolozin B (2004) Cholesterol and the biology of Alzheimer's disease. Neuron 41: 7-10.
