# Supplementary material for: Transcriptome Analysis of a Rotenone Model of Parkinsonism Reveals Complex I-Tied and -Untied Toxicity Mechanisms Common to Neurodegenerative Diseases
Source: PLoS One. 2012 Sep 7;7(9):e44700. doi: 10.1371/journal.pone.0044700 (PMC3436760; doi:10.1371/journal.pone.0044700)
Supplement: Table S4 — Neuroblastoma cells lineage specific markers. Format: PDF Size: 278 KB; This file can be viewed with: Adobe Acrobat Reader. (PDF) [file pone.0044700.s007.pdf]

Table S4. Neuroblastoma cells lineage specific markers

entrez ID	gene symbol	fold c. (1 week)		fold c. (4 weeks)		cell type (expression status)	refs.
		5 nM	50 nM	5 nM	50 nM		
2335	FN1	+3.6	+3.3	-1. 6	- 8.0	↑ S-type, ↓ N-type	[1, 2]
6285	S100B	+1.7	+1.5	-4.0	-2.4	↑ S-type, ↓ N-type	[2]
6282	S100A11	+1.8	+1.9	-5.6	-1.5	↑ S-type, ↓ N-type	[2]
6277	S100A6	+1.4	+1.5	-2.6	+1.1	↑ S-type, ↓ N-type	[2]
596	BCL2	+1.0	+1.3	-1.7	+2.2	↓ S-type, ↑ N-type	[3, 4]

Abbreviations: fold c. : fold change; refs: references; rotenone concentration in nanomolar

Notes: Emboldening indicates fold change is above 2. Official gene symbols are used at: (http://www.ncbi.nlm.nih.gov/gene/)

References

1. Ciccarone V, Spengler BA, Meyers MB, Biedler JL, Ross RA: **Phenotypic diversification in human neuroblastoma cells: expression of distinct neural crest lineages.** *Cancer Res* 1989, **49:**219-225.
2. Tsokos M, Scarpa S, Ross RA, Triche TJ: **Differentiation of human neuroblastoma recapitulates neural crest development. Study of morphology, neurotransmitter enzymes, and extracellular matrix proteins.** *Am J Pathol* 1987, **128:**484-496.
3. Lasorella A, Iavarone A, Israel MA: **Differentiation of neuroblastoma enhances Bcl-2 expression and induces alterations of apoptosis and drug resistance.** *Cancer Res* 1995, **55:**4711-4716.
4. Reed JC, Meister L, Tanaka S, Cuddy M, Yum S, Geyer C, Pleasure D: **Differential expression of bcl2 protooncogene in neuroblastoma and other human tumor cell lines of neural origin.** *Cancer Res* 1991, **51:**6529-6538.
