# Supplementary material for: Transcriptome Analysis of a Rotenone Model of Parkinsonism Reveals Complex I-Tied and -Untied Toxicity Mechanisms Common to Neurodegenerative Diseases
Source: PLoS One. 2012 Sep 7;7(9):e44700. doi: 10.1371/journal.pone.0044700 (PMC3436760; doi:10.1371/journal.pone.0044700)
Supplement: Table S5 — Greene et al.,(2008), [29] array data quality control and differentially-regulated genes (DRGs) analyzed using dCHIP. Format: PDF Size: 319 KB; This file can be viewed with: Adobe Acrobat Reader. (PDF) [file pone.0044700.s008.pdf]

Table S5. Greene et al.,(2008) array data quality control and differentially-regulated genes (DRGs)

treatment		array data quality control			number of DRGs [c, d, e]		
time (**weeks**)	rotenone (**nM**)	% present calls[a,b] (**mean ± sd**)	median intensity[a,b] (**mean ± sd**)	% single outliers (**mean ± sd**)	**up**	**down**	**total**
**1**	**0**[f]	49 ± 2	77 ± 0.0	0.13 ± 0.04	0	0	0
	**5**	49 ± 2	75 ± 6	0.17± 0.05	211	196	407
			above 2-fold change →		8 (4%)	8 (4%)	16 (4%)
**4**	**0**	49 ± 2	82 ± 9	0.14 ± 0.06	0	0	0
	**5**	49 ± 1	79 ± 6	0.13 ± 0.04	328	513	841
			above 2-fold change →		35 (11%)	25 (5%)	60 (7%)

Abbreviations: DRG: differentially-regulated gene. **Notes: a:** by dCHIP; **b:** no significant ($p < 0.05$) difference; **c:** by dCHIP analysis criteria: fold change > 1, $p < 0.05$; **d:** fold changes derived from this dataset were unusually low so the threshold was reduced to which is similar to only 2 control using only $p< 0.05$ as selection criterion as in the method used by Greene et al (2008); **e:** significance analysis of microarray (SAM), multiple test correction, FDR (%) 90th % tile < 1%, D-value> 1.2; **f:** only 2 cel files for this control time point were deposited by Greene et al (2008), at the GEO database by GSM107850 is missing.
